# Supplementary material for: Observational cohort study with internal and external validation of a predictive tool for identification of children in need of hospital admission from the emergency department: the Paediatric Admission Guidance in the Emergency Department (PAGE) score
Source: BMJ Open. 2020 Dec 31;10(12):e043864. doi: 10.1136/bmjopen-2020-043864 (PMC7780516; doi:10.1136/bmjopen-2020-043864)
Supplement: Supplementary data [file bmjopen-2020-043864supp001.pdf]

Statistical Appendix

This appendix contains only details not included in the main paper. It is intended that this Appendix will be made available online-only as a supplementary file.

Use of variables in the model

All of the variables were used in the model with the form of the individual variables and their suitability for inclusion in the model considered and adjusted as necessary.

Categorical variables with small categories

The category of behaviour ‘inappropriate’ was recorded for 54 children (<1%). This was too small a category to include in the modelling, and it was not clear what other category to amalgamate it with, as both ‘floppy’ and ‘listless’ could be considered ‘inappropriate’. These were marked as missing, removing those children from the analysis. The four categories of abnormal breathing (audible grunt, wheeze, stridor and tracheal tug) were very small (0 to 3%) and so breathing was re-categorised as ‘normal’ and ‘abnormal’. The category ‘severe recession’ was recorded for only 311 children over the three sites (between 0 and 1% per site), so ‘severe’ and ‘moderate’ recession were combined.

Continuous variables

During the regression modelling the fractional polynomial command made suggestions for the transformation of continuous variables. Age was included as reciprocal fractional polynomial of the original variable. Temperature and respiratory

Rowland A, *et al. BMJ Open* 2020; 10:e043864. doi: 10.1136/bmjopen-2020-043864

rate had a linear relationship with admittance and were included in the model as continuous variables.

For heart rate and oxygen saturation the plots of the fractional polynomials laid over the data were examined to visually assess how the models fit. These plots and the findings from the exploratory analysis jointly led decisions on the ultimate form of each of the variables.

- **Heart rate:** The fractional polynomial suggested a linear association with admittance. Examining the plot revealed that although a linear relationship is broadly appropriate it does a poor job of capturing the two large dips in probability of admittance. A more complex plot would have captured better the association between heart rate and admittance. With the large sample size such a curve could have been fitted without being overly concerned about overfitting. However, this would have defeated the overall aim of an easily used paper-based point scoring tool by requiring multiple coefficients in the final model. Heart rate was categorised as under 75, 75 to 125 and over 125.
- **Oxygen saturation:** The multivariable fractional polynomial modelling suggested that the raw oxygen saturation variable taken to the power of -2 was the best fitting form. On examination of the fractional polynomial plot it was clear that the plot fitted the data very well for oxygen saturations up to 90%, but above 90%, where 99% of the data lay, the polynomial failed to capture the more erratic association between saturation and admittance. Accordingly, a decision was taken to categorise the oxygen saturation variable based on the frequencies of the responses at each saturation

percentage, its observed relationship with admittance from the graph and on the previous PAT-POPS model (which aligns with clinical opinion of oxygen saturation cut-offs): 95%-100%; 90%-94%; <90%.

Being aware of the statistical implications of categorising continuous variables these decisions were not made lightly, however it was clear that no fractional polynomial would be able to capture the nature of the relationship better than categorisation.

Internal validation

Figure A shows the internal validation calibration plot. The blue ‘lowess’ line fitting the reference line so well is a demonstration of how well the model is fitting the data. Given the large sample size however this is to be expected. **Table A** shows the various internal validation results. The ‘apparent’ results are the average measures of validation for the 500 models created from the 500 bootstraps of the data. The ‘test’ results show the average performance of the models developed for each of those 500 bootstraps when applied to the original dataset. The ‘optimism’ results are the ‘apparent’ results subtracted from the ‘test’ results. ‘test’ results similar to ‘apparent’ results (and subsequently small ‘optimism’ results) suggest strong internal validity.

Rowland A, *et al. BMJ Open* 2020; 10:e043864. doi: 10.1136/bmjopen-2020-043864

Figure A. Calibration plot for internal validation.

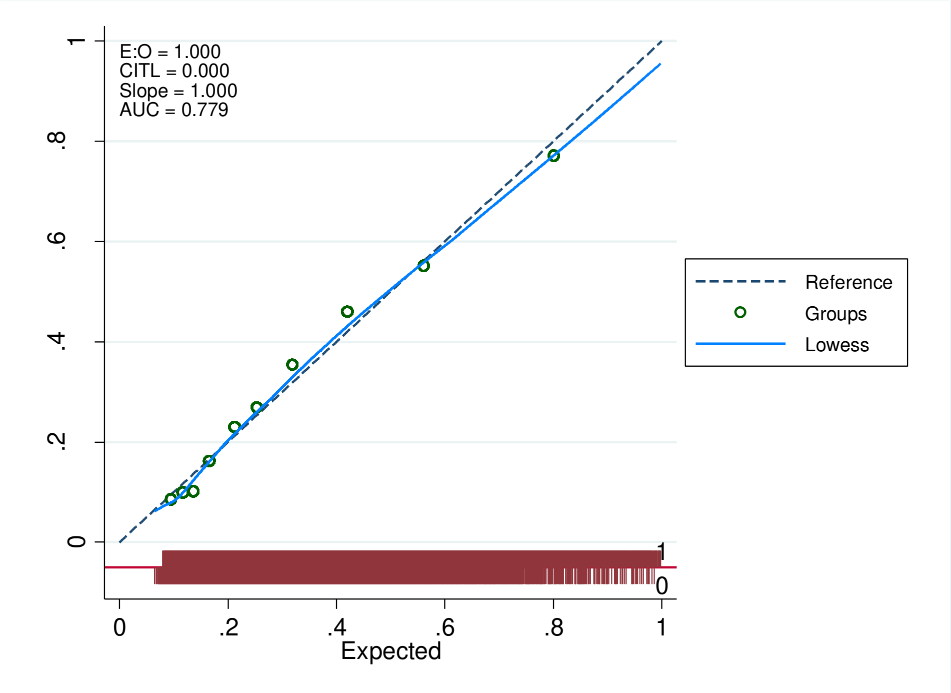

Table A. Results of internal validation.

Measure	Bootstraps	Mean	Standard deviation	Minimum	Maximum
**Apparent C-index**	500	.7790287	.0035788	.7685792	.7889659
**Apparent CITL**	500	0.00000000393	0.0000000181	- 0.000000103	0.000000190
**Apparent slope**	500	1	0.0000000549	1	1.000001
**Test C-index**	500	.7780273	.0005261	.7751072	.7791697
**Test CITL**	500	.0000289	.0158041	-.0458299	.0523462
**Test slope**	500	.9934335	.0175813	.9502897	1.043822
**Optimism C-index**	500	.0010013	.0035684	-.0097837	.0107504
**Optimism CITL**	500	-.0000289	.0158041	-.0523462	.0458299
**Optimism slope**	500	.0065665	.0175813	-.0438216	.0497103

Rowland A, *et al. BMJ Open* 2020; 10:e043864. doi: 10.1136/bmjopen-2020-043864

External validation: Site 2

Model reproducibility or transportability

The C-index for this membership model was 0.8078. The closer this number is to 1 the more likely it is that this represents model transportability rather than model reproducibility. So, in carrying out external validation using the Site 2 dataset an assessment is being made of whether the model works well in a different but related population. This was as expected given that from the exploratory analysis it could be seen that Site 2 represent a different mix of ethnicities as well as generally older and healthier patients.

Calibration and discrimination

The Brier score was lower in the Site 2 data (0.0653) than in the model development dataset, suggesting very good prediction accuracy in this external dataset. At 0.9864908 (0.896986 to 1.075996) the calibration slope was also close to the ideal of 1. Calibration-in-the-large (CITL) was less impressive at -1.218977 (-1.305886 to -1.132068). This suggests that the predicted probabilities were higher than the observed proportions. So, children in Site 2 were less likely to be admitted than the model predicted; a child with equivalent characteristics presenting at Site 1, for example, would be more likely to be admitted. This can be seen in the calibration plot **(Figure B)** where the observed probabilities are consistently lower than the expected probabilities, especially as the expected probabilities near 1. This was also evident in the E/O ratio of 2.7. This is the expected/observed ratio, how many were expected to be admitted based on the model versus how many actually were

admitted, so ideally this would be 1. That it is higher here suggests less people were admitted than would have been expected from the model. In terms of discrimination, the C-index was 0.7626 (0.74244 to 0.78279) **(Figure B)**.

Figure B. Calibration plot for Site 2 external validation.

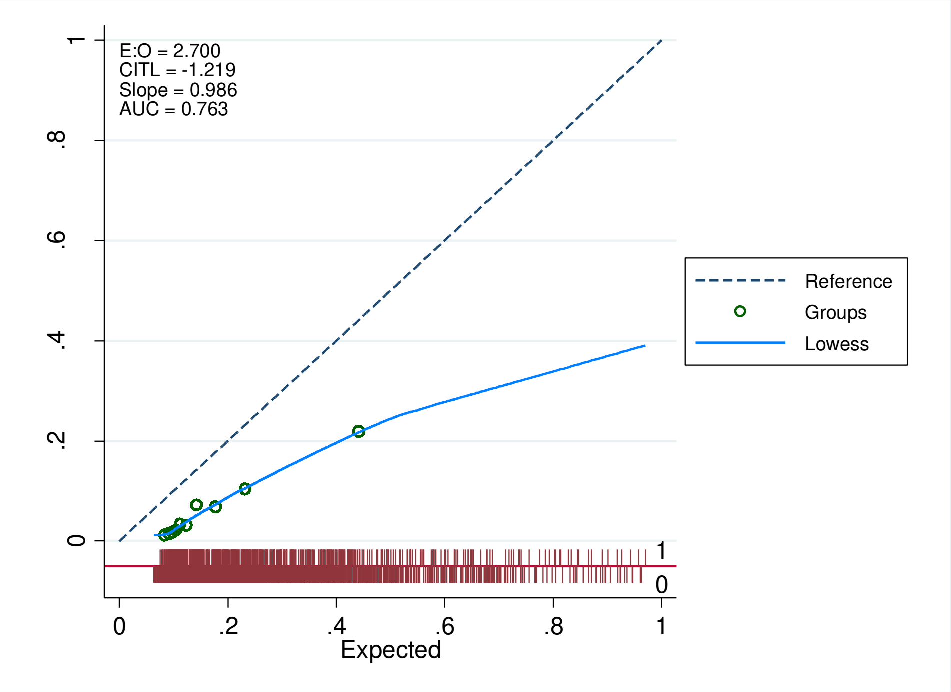

External validation: Site 3

Model reproducibility of transportability

The C-index for this membership model was 0.8154. This suggests that Site 1 and Site 3 are also quite different from each other in terms of their populations and therefore model transportability was assessed with the following results. That these two sites differ is expected; aside from the difference in ethnic make-up of the populations, Site 1 is an ED whereas Site 3 is an Urgent care centre and therefore sees healthier children.

Rowland A, *et al. BMJ Open* 2020; 10:e043864. doi: 10.1136/bmjopen-2020-043864

Calibration and discrimination

As with Site 2, when the model was applied to Site 3 the Brier score was a very low 0.0732. The calibration slope was over 1 at 1.075, this time suggesting overfitting, but as with Site 2 the CI also crossed 1 (0.976 to 1.123). The CITL was -1.055 (-1.123 to -0.987) suggesting predicted probabilities were higher than the observed proportions, again evident in **Figure C**. The E/O ratio was 2.3. The C-index was 0.7533 (0.734 to 0.770).

Figure C. Calibration plot for Site 3 external validation.

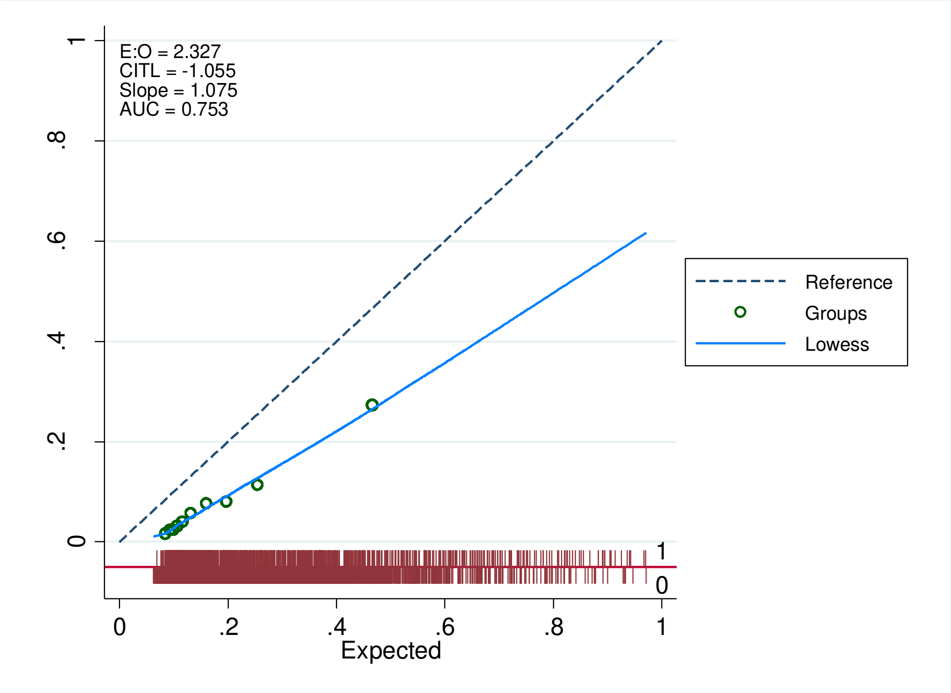

Overall

From these results it can be determined that the model transportability is good overall. With only slight over/under-fitting present at each of the external validation sites and C-indexes only modestly smaller than was found in the original model. It is

clear however that the model may over-estimate the probability of admission in external populations, probably owing to the admission rate at the development site (Site 1).
