# Supplementary material for: Observational cohort study with internal and external validation of a predictive tool for identification of children in need of hospital admission from the emergency department: the Paediatric Admission Guidance in the Emergency Department (PAGE) score
Source: BMJ Open. 2020 Dec 31;10(12):e043864. doi: 10.1136/bmjopen-2020-043864 (PMC7780516; doi:10.1136/bmjopen-2020-043864)
Supplement: Author's manuscript [file bmjopen-2020-043864.draft_revisions.pdf]

BMJ Open is committed to open peer review. As part of this commitment we make the peer review history of every article we publish publicly available.

When an article is published we post the peer reviewers' comments and the authors' responses online. We also post the versions of the paper that were used during peer review. These are the versions that the peer review comments apply to.

The versions of the paper that follow are the versions that were submitted during the peer review process. They are not the versions of record or the final published versions. They should not be cited or distributed as the published version of this manuscript.

BMJ Open is an open access journal and the full, final, typeset and author-corrected version of record of the manuscript is available on our site with no access controls, subscription charges or pay-per-view fees (http://bmjopen.bmj.com).

If you have any questions on BMJ Open's open peer review process please email info.bmjopen@bmj.com

BMJ Open

Development of a multivariable prediction model and scoring tool for identification of children in need of hospital admission from the emergency department: the Paediatric Admission Guidance in the Emergency Department (PAGE) score

Journal:	*BMJ Open*
Manuscript ID	bmjopen-2020-043864
Article Type:	Original research
Date Submitted by the Author:	17-Aug-2020

Complete List of Authors:	Rowland, Andrew; University of Salford<br>Cotterill, Sarah; The University of Manchester, Centre for Biostatistics<br>Heal, Calvin; The University of Manchester, Manchester Academic Health Science Centre; Salford Royal NHS Foundation Trust<br>Garratt, Natalie; Salford Royal Hospital<br>Long, Tony; University of Salford<br>Bonnett, Laura; University of Liverpool, Biostatistics<br>Brown, Stephen; Salford Royal Hospital<br>Woby, Steve ; Salford Royal NHS Foundation Trust,<br>Roland, Damian; University of Leicester, Health Sciences; University Hospitals of Leicester NHS Trust, Children's Emergency Department

Keywords:	Paediatric A&E and ambulatory care < PAEDIATRICS, Protocols & guidelines < HEALTH SERVICES ADMINISTRATION & MANAGEMENT, ACCIDENT & EMERGENCY MEDICINE

SCHOLARONE™
Manuscripts

I, the Submitting Author has the right to grant and does grant on behalf of all authors of the Work (as defined in the below author licence), an exclusive licence and/or a non-exclusive licence for contributions from authors who are: i) UK Crown employees; ii) where BMJ has agreed a CC-BY licence shall apply, and/or iii) in accordance with the terms applicable for US Federal Government officers or employees acting as part of their official duties; on a worldwide, perpetual, irrevocable, royalty-free basis to BMJ Publishing Group Ltd ("BMJ") its licensees and where the relevant Journal is co-owned by BMJ to the co-owners of the Journal, to publish the Work in this journal and any other BMJ products and to exploit all rights, as set out in our licence.

The Submitting Author accepts and understands that any supply made under these terms is made by BMJ to the Submitting Author unless you are acting as an employee on behalf of your employer or a postgraduate student of an affiliated institution which is paying any applicable article publishing charge ("APC") for Open Access articles. Where the Submitting Author wishes to make the Work available on an Open Access basis (and intends to pay the relevant APC), the terms of reuse of such Open Access shall be governed by a Creative Commons licence – details of these licences and which Creative Commons licence will apply to this Work are set out in our licence referred to above.

Other than as permitted in any relevant BMJ Author's Self Archiving Policies, I confirm this Work has not been accepted for publication elsewhere, is not being considered for publication elsewhere and does not duplicate material already published. I confirm all authors consent to publication of this Work and authorise the granting of this licence.

For peer review only

Development of a multivariable prediction model and scoring tool for identification of children in need of hospital admission from the emergency department: the **P**aediatric **A**dmission **G**uidance in the **E**mergency Department (**PAGE**) score

Rowland AG[1,2], Cotterill S[3], Heal C[3], Garratt N[4], Long T[1], Bonnett LJ[5], Brown S[4], Woby S, [1,4] and Roland D[6,7]

1 CYP@Salford, School of Health and Society, the University of Salford, Salford, UK

2 Emergency Department, North Manchester General Hospital, The Pennine Acute Hospitals NHS Trust, Manchester, UK

3 Centre for Biostatistics, School of Health Sciences, the University of Manchester, Manchester, UK

4 The Northern Care Alliance NHS Group, UK

5 Department of Biostatistics, University of Liverpool, UK

6 SAPHIRE Group, Health Sciences, University of Leicester, Leicester, UK

7 Paediatric Emergency Medicine Leicester Academic (PEMLA) Group, Children's Emergency Department, Leicester Royal Infirmary, Leicester, UK

Correspondence to

Dr. Damian Roland, Consultant in Children's Emergency Medicine, Children's Emergency Department, Leicester Royal Infirmary, Leicester, LE1 5WW

dr98@le.ac.uk

+44 (0)7727158213

For peer review only

Conflicts of Interest

None of the authors has anything to declare.

Contributions

Initial study design was by DR, AR, TL, SC, CH and SW with NG, LB and SB contributing to development. Study data was collected, processed and evaluated by SB, CH and SC. LB contributed to statistical analysis and review. NG managed the project under the leadership of DR and AR. All authors contributed to both initial and final drafts and agreed to the submission of the final draft.

Funding

This paper presents independent research funded by the National Institute for Health Research (NIHR) under its Research for Patient Benefit (RfPB) Programme (Grant Reference Number PB-PG-0815-20034). The views expressed are those of the author(s) and not necessarily those of the NIHR or the Department of Health and Social Care.

For peer review only

ABSTRACT

Objectives: To devise an assessment tool to aid discharge and admission decision-making in relation to children and young people in hospital urgent and emergency care facilities, and thereby improve the quality of care that patients receive, using a clinical prediction modelling approach

Design: Observational cohort study with internal and external validation of a predictive tool

Setting: Two general emergency departments and an urgent care centre in the North of England.

Participants: The eligibility criteria were children and young people 0-16 years of age who attended one of the three hospital sites within one NHS Trust. Children were excluded if they opted out of the study, were brought to the emergency department (ED) following their death in the community, or arrived in cardiac arrest when the heart rate and respiratory rate would be unmeasurable.

Main outcome measures: Admission or discharge. A participant was defined as being admitted to hospital if they left the ED to enter the hospital for further assessment, (including being admitted to an observation and assessment unit or hospital ward), either on first presentation or with the same complaint within seven days. Those who were not admitted were defined as having been discharged.

Results: The study collected data on 36365 participants. 15328 participants were included in the final analysis cohort (21045 observations) and 17710 participants were included in the validation cohort (23262 observations). There were 14 variables entered into the regression analysis. Of the 13 that remained in the final model, 10 were present in all 500 bootstraps. The resulting **P**aediatric **A**dmission **G**uidance in

For peer review only - http://bmjopen.bmj.com/site/about/guidelines.xhtml

the **E**mergency Department (PAGE) score demonstrated good internal validity. The C-index (AUROC) was 0.779 (95% CI 0.772 to 0.786).

Conclusions: For units without the immediate availability of paediatricians the PAGE score can assist staff to determine risk of admission. Cut off values will need to be adjusted to local circumstance.

Study protocol:

The study protocol has been published in an open access journal:

Riaz, S., Rowland, A., Woby, S. et al. Refining and testing the diagnostic accuracy of an assessment tool (PAT-POPS) to predict admission and discharge of children and young people who attend an emergency department: protocol for an observational study. BMC Pediatr 18, 303 (2018). https://doi.org/10.1186/s12887-018-1268-7

Trial registration: The protocol has been published and the study registered (NIHR RfPB Grant: PB-PG-0815-20034; ClinicalTrials.gov: 213469)

Strengths and limitations of this study'

- Pediatric early warning scores (PEWS) have utility in the detection of deterioration among inpatients but were not intended to identify children in the emergency department (ED) who need hospital admissions
- Based on data from children attending three non-tertiary (non-specialist) hospitals (n=36365) a clinical prediction model was developed and validated.
- The Pediatric Admission Guidance in the Emergency Department (PAGE) score, built from the clinical prediction model, is a practical tool for use in clinical settings and demonstrated good internal and external validity, with a C-index of 0·779 (95% CI 0·772 to 0·786).

For peer review only

- Admission rates varied between hospitals and while internal and external validation occurred the impact of prospective use to guide decision will need confirmation.

For peer review only

For peer review only - http://bmjopen.bmj.com/site/about/guidelines.xhtml

INTRODUCTION

Attendances at emergency departments (ED) by children and young people have risen year on year in the United Kingdom and are now in excess of 4 million per year **[1]**. The majority of these attendances are at services where the professional contact is by a practitioner without formal paediatric training. Given that a large proportion of these children and young people are discharged safely, the delivery of emergency and acute care does not rely on direct paediatric review.

However, systems are needed to recognise children who require further observation and investigation. Triage processes are well-established to determine the order in which patients should receive clinical review. Increasingly, a fuller assessment, including the patient's vital signs, is used to determine an acuity score to define risk of illness or deterioration. These scores, often termed early warning scores (EWS), originated in adult in-patient environments where it was recognised that death or intensive care admission was often preceded by derangement of the patient's physiology **[2]**. These findings led to the development of paediatric early warning scores (PEWS) which have been shown to have utility in the detection of deterioration, but not always in the prevention of mortality **[3]**. The face validity of such scores led to widespread utilisation and attempts to employ them in different emergency and pre-hospital environments from those where the scores were devised. Initial reviews demonstrated relatively poor performance **[4]**, in turn leading to the development of bespoke scores validated in emergency departments themselves.

For peer review only - http://bmjopen.bmj.com/site/about/guidelines.xhtml

There is no existing gold standard outcome measure for the decision to admit or discharge a child or young person from the ED **[5]**, and the decision to admit is a complex one, which can vary between clinicians and hospitals. One early warning scoring system, the Paediatric Observation Priority Score (POPS), recommended by the Intercollegiate Committee for Standards for Children and Young People in Emergency Care Settings **[6]** has shown initial promise in aiding recognition of unwell children but also aiding safe discharge decisions **[7,8]**.

A modified version of POPS, developed at The Pennine Acute Hospitals NHS Trust (PAT-POPS), has been shown to have greater discriminatory power than an inpatient PEWS **[9]**. The majority of EWS systems are derived and validated at large teaching institutions ("tertiary centres") and so may not be applicable outside tertiary centres **[10]**. In this study PAT-POPS was used as the initial starting point to derive and validate a score to aid disposition decisions in non-tertiary centres. This would have external validity in the majority of locations where children and young people are assessed in the United Kingdom and potentially beyond.

METHODS

A published protocol is available **[11]** and only the core components of the methods are summarised in this paper. In this paper "participant" means a patient recruited into the study at one of the study sites.

Patient And Public Involvement

The study was supported by a patient advisory group which provided input to the programme of research. This patient advisory group met with author TL during the

For peer review only - http://bmjopen.bmj.com/site/about/guidelines.xhtml

study. Patients partnered with the study team for the design of the study, the informational material to support the opt-out consent process. At the end of the study twenty-one parents were consulted in one of the EDs on how the cut point might be made. Most found this a particularly difficult task, hovering around the centre of the scale. When the notion was introduced of a supporting community service that would know about their child and for which they had a contact number if worried, a remarkable difference was observed. All but two (both fathers) considered correct discharge to be more important than correct admission, and, overall, most thought that about 60-70% correct discharge was optimal. Two mothers would be sufficiently confident for a much higher specificity, being prepared to discharge 90-95% patients correctly.

Sample Population

The study population was recruited consecutively. Data collection was prospective over a whole year (1 March 2018 to 28 February 2019) to avoid the effects of bias from seasonal variability. The eligibility criteria were children and young people 0-16 years of age who attended one of three hospital sites within one NHS Trust in Greater Manchester, UK. Children were excluded if they opted out of the study, were brought to the emergency department (ED) following their death in the community, or arrived in cardiac arrest when the heart rate and respiratory rate would be unmeasurable.

For peer review only - http://bmjopen.bmj.com/site/about/guidelines.xhtml

Admission

A participant was defined as being admitted to hospital if they left the ED to enter the hospital for further assessment, (including observation and assessment unit or hospital ward), either on first presentation or with the same complaint within seven days. This was approved by the study patient and public involvement (PPI) group, which saw admission and discharge in such terms, and by discussion with three ED doctors.

The decision to admit the participant was made by a clinician (either a doctor or a nurse practitioner). They followed existing guidelines, using usual methods of clinical judgement, and were blinded to the score as this had not yet been derived at the point of participant assessment (but had access to all vital signs and observations normally collected). Admission data from all three hospitals was accessed from existing NHS trust electronic systems.

Predictors

All of the variables in the modified PAT-POPS plus additional variables included in other scores were considered for inclusion in the PAGE score (**Table 1**). Other data collection included reason for attendance at the ED, diagnosis, death in the ED, children leaving the ED before admission decision, children's characteristics (age, gender, and ethnicity), investigated deaths and serious incidents.

[INSERT TABLE 1 HERE]

For peer review only

Sample Size

In the protocol it was estimated that 9000 children were needed for the development of the prediction model and 7000 children in the independent validation. The minimum sample size needed to do both analyses was therefore estimated as 16000 children. More data than needed was allowed for (and this approach was granted at ethics review) due to the need to collect data for a full year to capture seasonal variation in childhood illness and injury. Intermittent data collection would not help implementation of the tool and would have required the employment of specific staff for the project, which would have been significantly more costly.

Analysis

Analysis was conducted in StataMP 14 **[12]** using two-sided 95% confidence intervals and the 5% significance level. Analysis was reported according to the TRIPOD **[13]** and STARD **[14]** reporting guidelines. A flow diagram summarises participant opt-out, recruitment and data collection, by site (**Figure 1**).

[INSERT FIGURE 1 HERE]

Final analysis was undertaken after all data had been entered into the database, and the database had been cleaned and locked.

Children were excluded from the analysis if the outcome variable (admission) was missing or if all the independent variables were missing. For variables with over 4% rates of missingness data was imputed using hot-deck imputation **[15]**. This involves stratifying participants by key predictive variables (injury/illness status, admission

For peer review only - http://bmjopen.bmj.com/site/about/guidelines.xhtml

status and age) and replacing missing values with those of a participant from the same strata.

Participants were described with respect to the variables in the model, both overall and by site, reported as number (%) for categorical variables; mean (standard deviation (sd), min, max) for normally distributed variables; median (inter-quartile range (IQR), min, max) for other numeric variables.

Stage 1: Model development

Children from one hospital site were utilised for the model development. Logistic regression models were developed with hospital admission as the outcome and including all candidate variables. Due to several of the variables being non-linearly associated with the outcome a closed test procedure was used to determine the best functional form of each continuous variable and concurrently whether it should be included. This involved starting with a model that included all potential predictors and testing the best fitting fractional polynomial form of each continuous variable individually **[16]**. Variables were removed from the model according to p-values with any less than 0.10 being excluded. For categorical variables, if at least one category was significant all were included initially. Quality of the data and risk of bias was assessed using PROBAST **[17]**.

Stage 2: Internal validation

The calibration score and calibration slope were examined to assess how well the predictions from the model matched the data and calibration plots were used to compare agreement between predicted and observed injury and illness.

For peer review only - http://bmjopen.bmj.com/site/about/guidelines.xhtml

Discrimination was also considered, to measure how well the model separated between individuals who were admitted and those who were not (C-statistic, which is equivalent to area under the ROC curve).

The Brier score has been reported. Internal validation was performed by applying the original model to 500 bootstrapped samples. The discrimination and calibration performance of the model in each of the bootstrap samples was compared with the model fitted to the original data to provide a measure of optimism. Due to the large sample size, it was not necessary to adjust for the optimism **[18]**. The inclusion/exclusion of any predictors which featured in the selected model but only rarely across the bootstrap samples (or vice versa) was noted.

The output of Stage 1 and 2 was the Paediatric Admission Guidance in the Emergency Department (PAGE) score which aimed to predict hospital admission, based on the weight of each predictor.

Stage 3: External validation

Assessing model performance

External validation was undertaken using data from two other hospitals. The developed model was applied to each external dataset, and calibration and discrimination measures were reported, as above.

Recalibration

After looking at both the development and the external datasets, and with clinical opinion, consideration was given to which dataset most closely represents the

For peer review only - http://bmjopen.bmj.com/site/about/guidelines.xhtml

majority of UK emergency departments at which children attend, and accordingly whether or not to recalibrate the model based on one of the external validation datasets.

Stage 4: Developing a point scoring system

The model's regression coefficients were used to assign integer points to each level of each risk factor, and a reference table of risk per possible points total was produced, following established guidelines **[19]**. Together these provide a clinically useful score. By applying the points scores to the development dataset it was possible to calculate the sensitivity, specificity, positive and negative likelihood ratios of PAGE (index test) in predicting admission (reference test) with 95% confidence intervals. A cross tabulation of the results of the index test by the results of the reference test was reported, including indeterminate and missing results.

Consensus Meeting

A meeting was held to examine the statistical data, and to agree which cut points of the PAGE score were most suitable to predict (i) safe admission decision and (ii) safe discharge decision, including consideration of what weight to give to sensitivity and specificity in making the decision. All of our research team, plus paediatric ED clinicians and an independent methodologist, were invited to attend.

Stage 5: Further analyses

The usefulness of the PAGE score was assessed by calculating the sensitivity, specificity, positive and negative likelihood ratios at the chosen cut-points, to predict admission and discharge.

For peer review only - http://bmjopen.bmj.com/site/about/guidelines.xhtml

Ethics and Research Governance

The West Midlands Research Ethics Committee approved this project (**17/WM/0436**) on the 20th December 2017. An opt-out consent design was approved and as such there were no plans to disseminate the results to participants.

For peer review only

RESULTS

The unit of analysis was a visit by a child to the ED. **Figure 1** shows the numbers and reasons for exclusions of visits (and participants) from the dataset. Children were omitted because they opted out of the study (n=4, Site 1), died in the ED (n=8, Site 1) or left the ED before an admission decision was made (n=892, Site 1). Duplicate entries where participants had more than one entry with the same date and time (n=199, Site 1) were deleted.

Missing visits

There were two types of visit that were excluded completely from the development dataset due to extensive missing data. The first (marked as 'blanks' in **Figure 1**) were missing all data except for patient demographics. After data collection was completed, we discovered that Site 1 had a two-tier system which funnels off particularly 'well' children into a separate nurse-led service where no further study information was recorded (n=3865, Site 1). The second were visits where data was missing for enough clinical measures and judgements to prevent imputation and inclusion in the analysis This was likely due to them being measurements that would not routinely be taken for certain types of attendances, such as minor injuries (n=421, Site 1).

Missing variables

Most variables had very low rates of missingness (0 to 2%) and no imputation was required. Four variables: heart rate, temperature, respiratory rates and oxygen saturation had higher rates, which varied by site: Site 1 (8-11%), Site 2 (2-4%) and

For peer review only - http://bmjopen.bmj.com/site/about/guidelines.xhtml

Site 3 (1-2%). Reasons for the missingness, and differences in missingness by site, are not clear. Values were imputed using hot-deck imputation **[15]**.

Descriptive statistics / summary of population

Table 1 summarises the demographic and medical characteristics of the sample population at each site. This comprises all participants who met the inclusion criteria. The admission rate of Site 1 (the development dataset) was 32% compared to 6% at Site 2 and 8% at Site 3. Compared to the external validation sites, children in the development dataset were younger, showed signs of being more unwell (higher heart rate, more prevalence of recession and breathing abnormalities) and were more likely to show cause for concern for the nurses. Temperature, oxygen saturation, responsiveness and behaviour were very similar across the sites.

The form of variables and their suitability for inclusion in the model were considered and adjusted as necessary. Categorical variables were re-coded to avoid any small categories. Abnormal breathing was re-categorised as 'normal' and 'abnormal'. 'Severe' and 'moderate' recession were amalgamated. Two continuous variables (heart rate and oxygen saturation) were categorised as in their continuous form we were unable to model them adequately whilst keeping the final score readily usable in clinical practice.

Regression results

The final multivariable logistic regression model is shown in **Table 2**.

Responsiveness was omitted from the final model, because once the other variables were accounted for, it had no effect on admission. All other variables were retained

For peer review only - http://bmjopen.bmj.com/site/about/guidelines.xhtml

in the final model and had a statistically significant relationship with admission except for the ‘under 75 bpm’ heart rate category.

[INSERT TABLE 2 HERE]

The younger the child the more likely admission is and the effect is much stronger for much younger participants. Compared to those aged 4 to 16 years old, those aged 0-1 months had an increase in admission odds of 1.880 (1.876 to 1.885). Low oxygen saturation was the strongest predictor of admission in the study and Nurse judgement was strongly associated with admission. Variables that were introduced in this study as additional to the existing PAT-POPS score were multi-morbidity, (1.94 (1.60 to 2.35)), arrival by ambulance (2.42 (2.22 to 2.63)) and being advised to attend by a medical professional (2.16 (1.97 to 2.36)).

Internal Validation

As would be expected from such a large sample size, the model demonstrated good internal validity. The discrimination (C-index) of the model in the original dataset was 0.779 (95% CI 0.772 to 0.786) and the mean in the bootstrap samples was 0.779 (95% CI 0.767 to 0.789) suggesting no model optimism. Full calibration and discrimination results are reported in the supplementary file.

Calibration

The Brier score was 0.1664, the calibration slope was 1.000 and calibration-in-the-large was -0.000. The calibration plot indicates that predictions from the model

For peer review only - http://bmjopen.bmj.com/site/about/guidelines.xhtml

match the data very well for low to moderate risk individuals. Above this, the model slightly over-predicts the probability of admission.

External validation

The external validation was undertaken in each of the two external datasets separately (**Table 3**).

[INSERT TABLE 3 HERE]

These results indicate that the model transportability is good overall, with only slight over/under-fitting present at each of the external validation sites and C-indexes (0.763 and 753 for site 2 and 3 respectively), only modestly smaller than were found in the original model. It is clear however that the model may over-estimate the probability of admission in external populations, probably owing to the unusually high admission rate of the development site (site 1).

Of the three sites, site 1 is most typical of United Kingdom EDs in respect of annual attendances and size of unit. This being the case, recalibration of the model so it performed better in the two external datasets was not necessary. The original model was carried forward to the stage of developing the point scoring system.

Derivation of <u>P</u>aediatric <u>A</u>dmission <u>G</u>uidance in the <u>E</u>mergency Department (PAGE) score

The PAGE score has a minimum value of 0 and a maximum of 30. The characteristic that yields the largest numbers of points is having a lower than normal oxygen

For peer review only - http://bmjopen.bmj.com/site/about/guidelines.xhtml

saturation, worth 4 or 5 points depending on severity. Age, against clinical expectation, seemed only predictive of admission when the participant was younger than 6 months.

[INSERT TABLE 4 HERE]

The sensitivity and specificity of each potential cut-point of the tool is shown in **table 5**. As the development of the point-scoring system was effectively a simplification of the regression model, we expected a relative decrease in its predictive power. The difference in the discrimination of the regression model and the resulting point scoring tool however proved to be minimal (**Figure 2**): a drop from 0.779 (95% CI 0.772 to 785) to 0.775 (95% CI 0.768 to 0.781).

[INSERT TABLE 5 HERE]

[INSERT FIGURE 2 HERE]

The cut-point for PAGE that most closely aligns with the sensitivity and specificity of the chosen original PAT-POPS cut-off is a score of 7 or more. This yields a sensitivity of 48.21% and specificity of 87.61%. Based on the preferences of those at the consensus meeting the chosen cut-off for their trusts would be either 6 or 7 points on the basis that consensus meeting attendees indicated a preference for giving more weight to higher specificity (ensuring the right children were discharged) than sensitivity. It is, of course, a matter for health care organisations to determine

For peer review only - http://bmjopen.bmj.com/site/about/guidelines.xhtml

their own cut-off based on local service arrangements, however it is suggested that a cut-off of 6 or 7 may be appropriate.

DISCUSSION

In this study of over 30000 children the **P**aediatric **A**dmission **G**uidance in the **E**mergency Department (PAGE) score was derived. This is the first study of its kind in non-tertiary emergency departments and therefore should have good external validity in the majority of locations in which children are treated and in which paediatricians are not immediately available. While the development site (site 1), a type 1 emergency department with trauma unit status, had a high admission rate, it is noted that the national average is 13.8% (confidence intervals unavailable) **[20]**. At site 1 the participants presenting with minor injury are streamed into an emergency nurse practitioner (ENP) stream which artificially increases the apparent admission rate. When tested on the sites with admission rates of 6% and 8% the score still performed well, highlighting that the score may have utility in organisations with a range of admission rates. The area under the ROC curve 0.779 (95% CI 0.772 to 0.785) performing better than any other previously published similar tool **[21].**

The PAGE score should not be considered a typical early warning score as it has been derived for the sole purpose of determining risk of admission, rather than risk of deterioration. A number of the components are temporally fixed (i.e. arrival by ambulance and referred by health care professional) and will not change, so the PAGE score is not a sequential scoring tool, either. However, as all the components are commonly utilised it provides an easy to measure score at initial assessment that can guide the emergency pathway utilisation for the child or young person. It is

For peer review only - http://bmjopen.bmj.com/site/about/guidelines.xhtml

important to note that age is a component of the score itself (i.e. there are not separate scores for each age group). For those used to traditional EWS approaches this may cause uncertainty as the heart rate cut off appears low for many young infants (>120 gives a score of 1 across all ages). However, it is important to remember that this is a population-derived tool. Importantly, it will be necessary for local departments to derive their own cut points as where they are set depends on what the local services can offer both in the hospital and in the community.

The derivation of score revealed some interesting results in relation to the relative importance of the different components. While nurse judgement was the categorical variable with the strongest association with admission this was only marginal. The confidence interval for ‘floppy’ means that this may also be a strong predictor, but there were insufficient numbers of participants presenting with this characteristic to be able to demonstrate this. Although from a continuous variable, having an oxygen saturation below 90 was a much stronger predictor of admittance, which is in keeping with known clinical practice. However, in a group of objective measures it is perhaps surprising one of the most subjective, judgement, was one of the strongest associations. This may reflect the important predictive information that cannot or will not be obtained through clinical measurements and is clearly an area worthy of further study.

The PAGE score did not assess individual illness categories (it was not designed to highlight risk of sepsis) nor did it determine length of stay. Both of these can be determined in future prospective observational cohorts.

For peer review only

LIMITATIONS

There are limitations to the study design. Admitting or discharging a participant based on the PAGE score is only as appropriate as the original judgement of the clinicians on which the score is based. That is to say we do not know what proportion of admission decisions ultimately prove beneficial to the participant. This is not a limitation specific to our study but a wider consideration for any score that predicts admission. A number of factors influence the decision to admit a participant, and while we have attempted to determine all relevant components there may be socio-cultural factors which were missed in the initial derivation work.

CONCLUSION

For units without the immediate availability of paediatricians the PAGE score can assist staff to determine risk of admission. Cut off values will need to be adjusted to local circumstances.

REFERENCES

1. NHS Digital. https://digital.nhs.uk/data-and-information/publications/statistical/hospital-accident--emergency-activity/2018-19 Website last accessed 28th March 2020
2. NICE. Acutely Ill Patients in Hospital: Recognition and Response to Acute Illness in Adults in Hospitals, 2007
3. Roland D. Beyond Mortality: Improving outcomes for children who deteriorate in inpatient settings. J. Hosp. Med. 2019 August;14(8):512-513
4. Seiger N, Maconochie I, Oostenbrink R,Moll HA. Validity of different pediatric early warning scores in the emergency department Pediatrics 2013; 132
5. Lillitos PJ, Lyttle MD, Roland D On behalf of PERUKI and GAPRUKI, et al Defining significant childhood illness and injury in the Emergency Department: a consensus of UK and Ireland expert opinion Emergency Medicine Journal 2018;35:685-690
6. RCPCH. Standards for children and young people in emergency care settings 2018

For peer review only - http://bmjopen.bmj.com/site/about/guidelines.xhtml

7. Roland D, Lewis G, Fielding P, Hakim C, Watts A and Davies F. The Paediatric Observation Priority Score: A System to Aid Detection of Serious Illness and Assist in Safe Discharge Open Journal of Emergency Medicine 2016 4(2) 38-44
8. Roland D, Arshad F, Coats T, and Davies F. Baseline Characteristics of the Paediatric Observation Priority Score in Emergency Departments outside Its Centre of Derivation BioMed Research International, vol. 2017, Article ID 9060852, 5 pages, 2017. doi:10.1155/2017/9060852
9. Cotterill, S, Rowland, AG, Kelly, J, Lees, H and Kamara, M 2016, 'Diagnostic accuracy of PAT-POPS and ManChEWS for admissions of children from the emergency department', Emergency Medicine Journal, 33 (11)
10. Challen K and Roland D. Early Warning Scores: a health warning EMJ 2016, 33(11):812-817
11. Riaz, S, Rowland, AG, Woby, SR, Long, T, Livesley, J, Cotterill, S, Heal, C and Roland, D 2018, 'Refining and testing the diagnostic accuracy of an assessment tool (PAT-POPS) to predict admission and discharge of children and young people who attend an emergency department : protocol for an observational study' , BMC Pediatrics, 18 (303)
12. StataCorp. 2015. Stata Statistical Software: Release 14. College Station, TX: StataCorp LP.
13. Moons KG, Altman DG, Reitsma JB, et al. Transparent Reporting of a multivariable prediction model for Individual Prognosis Or Diagnosis (TRIPOD): Explanation and Elaboration. Ann Intern Med. 2015;162:W1–W73. doi: https://doi.org/10.7326/M14-0698
14. Cohen JF, Korevaar DA, Altman DG, et al STARD 2015 guidelines for reporting diagnostic accuracy studies: explanation and elaboration BMJ Open 2016;6:e012799. doi: 10.1136/bmjopen-2016-012799
15. Kalton, G., & Kish, L. (1981). Two efficient random imputation procedures. In Proceedings of the survey research methods section (pp. 146-151). American Statistical Association
16. Royston P, Sauerbrei W. Multivariable model-building: a pragmatic approach to regression anaylsis based on fractional polynomials for modelling continuous variables. John Wiley & Sons; 2008 Sep 15
17. Wolff RF, Moons KG, Riley RD, et al, for the PROBAST Group†. PROBAST: A Tool to Assess the Risk of Bias and Applicability of Prediction Model Studies. Ann Intern Med. 2019;170:51–58. doi: https://doi.org/10.7326/M18-1376
18. Riley, R. D., Ensor, J., Snell, K. I., Debray, T. P., Altman, D. G., Moons, K. G., & Collins, G. S. (2016). External validation of clinical prediction models using big datasets from e-health records or IPD meta-analysis: opportunities and challenges. bmj, 353, i3140
19. Sullivan, L.M., Massaro, J.M. and D'Agostino Sr, R.B., 2004. Presentation of multivariate data for clinical use: The Framingham Study risk score functions. Statistics in medicine, 23(10), pp.1631-1660.
20. This data was obtained via a Freedom of Information request to NHS Digital but did not provide confidence intervals or a range
21. Seiger N, Maconochie I, Oostenbrink R, Moll HA. Validity of different pediatric early warning scores in the emergency department. Pediatrics. 2013;132(4):e841–e850. doi:10.1542/peds.2012-3594

For peer review only - http://bmjopen.bmj.com/site/about/guidelines.xhtml

Table 1: Characteristics of the sample population at each site (who met the inclusion criteria)

	**Site 1 (Emergency Department) (development dataset) Visits = 21480 Participants = 15484**			**Site 2 (Emergency Department) Visits = 9972 Participants = 7817**			**Site 3 (Urgent Care Centre) Visits = 13429 Participants = 9971**		
Variable	Summary	Min/Max	Missing (%)	Summary	Min/Max	Missing (%)	Summary	Min/Max	Missing (%)
**Age** in years (median, IQR)	3 (1 to 8)	0/15	0	7 (3 to 12)	0/15	0	6 (2 to 10)	0/15	0
**Gender**						0			0
Female	9384 (44%)		0	4486 (45%)			6131 (54%)		
**Ethnicity**			0			0			0
White British	11162 (52%)			7081 (71%)			7046 (52%)		
Irish + Gypsy or Irish Traveller	34 (0%)			26 (0%)			61 (0%)		
Other White	569 (3%)			298 (3%)			369 (3%)		
White and Black Caribbean	158 (1%)			89 (1%)			51 (0%)		
White and Black African	121 (1%)			53 (1%)			80 (1%)		
White and Asian	255 (1%)			118 (1%)			217 (2%)		
Other Mixed	294 (1%)			107 (1%)			244 (2%)		
Indian	75 (0%)			45 (0%)			51 (0%)		
Pakistani	4245 (20%)			1165 (12%)			61 (0%)		
Bangladeshi	2060 (10%)			36 (0%			524 (4%)		
Chinese	46 (0%)			42 (0%)			29 (0%)		
Other Asian + Asian/Asian British	302 (1%)			202 (2%)			213 (2%)		
African									
Caribbean	374 (2%)			114 (1%)			242 (2%)		
Other Black +	24 (0%)			8 (0%)			21 (0%)		
Black/African/Caribbean/Black	149 (1%)			54 (1%)			168 (1%)		
British									
Unknown / not stated	1010 (5%)			270 (3%)			169 (1%)		
**Heart rate** (mean /sd)	121.6 (30.5)		11	104.5 (26.1)		4	109.7 (27.0)		2
**Temperature** (median /iqr)	36.9 (36.5 to 37.4)	32.6/41.7	10	36.7 (36.4 to 37.1)	34.4/41.1	3	36.7 (36.4 to 37.1)	35.0/41.2	1

For peer review only - http://bmjopen.bmj.com/site/about/guidelines.xhtml

	Site 1 (Emergency Department) (development dataset) Visits = 21480 Participants = 15484			Site 2 (Emergency Department) Visits = 9972 Participants = 7817			Site 3 (Urgent Care Centre) Visits = 13429 Participants = 9971		
**Respiratory rate**	26 (22 to 32)	10/90**	10	22 (20 to 26)	12/88	3	24 (20 to 26)	12/80	2
**Oxygen saturation**	99 (98 to 100)	63/100	10	99 (98 to 100)	70/100	4	99 (99 to 100)	67/100	2
**Requires supplement oxygen**	386 (2%)		0	31 (0%)		0	99 (1%)		0
**Breathing**			2			0			0
None of the above	19678 (93%)			9713 (98%)			13038 (98%)		
Audible grunt	52 (0%)			9 (0%)			25 (0%)		
Wheeze	709 (3%)			135 (1%)			190 (1%)		
Stridor	52 (0%)			6 (0%)			9 (0%)		
Tracheal tug	624 (3%)			68 (1%)			102 (1%)		
**Recession**			0			0			0
No recession	18757 (87%)			9748 (98%)			12900 (96%)		
Mild recession	1764 (8%)			153 (2%)			338 (3%)		
Moderate	743 (3%)			43 (0%)			114 (1%)		
Severe	213 (1%)			27 (0%)			75 (1%)		
**Responsiveness**			0			0			0
Alert	21044 (98%)			9775 (98%)			13252 (99%)		
Responds to pain	53 (0%)			10 (0%)			5 (0%)		
Responds to voice	126 (1%)			15 (0%)			19 (0%)		
Unresponsive	253 (1%)			171 (2%)			151 (1%)		
**Nurse Judgement**			0			0			0
No concern	6476 (30%)			6894 (69%)			9888 (74%)		
Low level concern	11645 (54%)			2756 (28%)			3268 (24%)		
Child looks unwell/high concern	3355 (16%)			321 (3%)			271 (2%)		
**Behaviour**			0			0			0
Normal for age	20349 (95%)			9720 (97%)			13105 (98%)		
Agitated	356 (2%)			105 (1%)			121 (1%)		
Floppy	138 (1%)			22 (0%)			23 (0%)		
Inappropriate	54 (0%)			9 (0%)			8 (0%)		
Listless	579 (3%)			115 (1%)			170 (1%)		

For peer review only

	**Site 1 (Emergency Department) (development dataset) Visits = 21480 Participants = 15484**			**Site 2 (Emergency Department) Visits = 9972 Participants = 7817**			**Site 3 (Urgent Care Centre) Visits = 13429 Participants = 9971**		
Multi-morbidity	749 (3%)		0	148 (1%)		0	551 (4%)		0
**Arrived by ambulance**	4539 (21%)		0	20 (0%)		0	50 (0%)		0
**Admitted now or next 7 days**	6810 (32%)		0	600 (6%)		0	1009 (8%)		0

For peer review only

For peer review only - http://bmjopen.bmj.com/site/about/guidelines.xhtml

Table 2: Final regression model

Variable	OR	95% CI low	95% CI high	P
Age				<0.001
0-1 months	1.880	1.876	1.885	
2-5 months	1.279	1.276	1.282	
6-11 months	1.125	1.122	1.282	
1-3 years	1.031	1.029	1.034	
4-16 years	Reference			
Heart rate				
<75	.959	.807	1.141	0.638
75-125	Reference			
>125	1.422	1.303	1.552	<0.001
Temperature (degrees Celsius)				<0.001
<38	Reference			
38-39	1.365	1.307	1.424	
>39	1.763	1.689	1.840	
Respiratory rate	1.029	1.023	1.035	<0.001
Oxygen saturation				<0.001
95-100	Reference			
90-94	3.381	2.654	4.305	
<90	5.477	2.953	10.157	
Requires supplementary oxygen	1.872	1.437	2.440	<0.001
Breathing				
Normal	Reference			
Abnormal	1.681	1.423	1.985	<0.001
Recession				
None	Reference			
Mild	1.281	1.117	1.469	<0.001
Moderate/Severe	1.484	1.183	1.863	0.001
Behaviour category				
Normal	Reference			
Agitated	1.353	1.043	1.754	0.023
Floppy	2.046	1.371	3.054	<0.001
Listless	1.498	1.217	1.843	<0.001
Nurse judgement				
No concern	Reference			
Low level concern	1.361	1.258	1.473	<0.001
High concern	2.677	2.388	3.002	<0.001
Multimorbidity	1.937	1.599	2.346	<0.001
Arrived by ambulance	2.416	2.223	2.625	<0.001
Advised by medical professional	2.159	1.975	2.361	<0.001
Constant	.144	0.133	0.156	<0.001

For peer review only - http://bmjopen.bmj.com/site/about/guidelines.xhtml

Table 3: external validation results

Site	Briers score	Calibration slope	CITL	E/O	C-index
2	0.065	0.986<br>(0.897 to 1.076)	-1.219<br>(-1.306 to -1.132)	2.673	0.763<br>(0.742 to 0.783)
3	0.073	1.075<br>(1.002 to 1.149)	-1.055<br>(-1.123 to -.987)	2.275	0.753<br>(0.737 to 0.770)

For peer review only

For peer review only - http://bmjopen.bmj.com/site/about/guidelines.xhtml

Table 4: PAGE point scoring tool

Variable	Category	Point(s)
Age (months)	0-1	2
	2-5	1
	≥ 6	0
Heart rate (BPM)	≤ 125 (ie 0-125)	0
	>125 (ie 126 and above)	1
Respiratory rate	0-25	0
	26-60	2
	>60	3
Temperature (degrees Celsius)	<38	0
	38-39	1
	>39	2
Oxygen saturation (%)	>94	0
	90-94	4
	<90	5
Requires supplementary oxygen	No	0
	Yes	2
Breathing	Normal	0
	Abnormal	2
Recession	No recession	0
	Any recession	1
Behaviour	Normal	0
	Agitated or Listless	1
	Floppy	2
Nurse Judgement	No concern	0
	Low level concern	1
	High concern	3
Multi-morbidity	No	0
	Yes	2
Arrived by ambulance	No	0
	Yes	3
Advised by medical professional to attend	No	0
	Yes	2

For peer review only - http://bmjopen.bmj.com/site/about/guidelines.xhtml

Table 5: Sensitivity and specificity for each cut-point of PAGE

Cut-point for admission decision	Sensitivity (95% CI)	Specificity (95% CI)	Likelihood ratio +	Likelihood ratio -
≥0	100%	0%	1.0000	-
≥1	96.83 (96.38 to 97.23)	15.80 (15.22 to 16.41)	1.1501	0.2010
≥2	91.13 (90.42 to 91.79)	36.34 (35.56 to 37.13)	1.4315	0.2441
≥3	87.70 (86.89 to 88.46)	45.84 (45.03 to 46.65)	1.6192	0.2684
≥4	78.87 (77.90 to 79.81)	61.03 (62.23 to 63.82)	2.1330	0.3353
≥5	68.61 (67.49 to 69.70)	73.69 (72.97 to 74.40)	2.6074	0.4261
≥6	59.47 (58.29 to 60.64)	80.86 (80.21 to 81.49)	3.1070	0.5012
≥7	48.56 (47.37 to 49.76)	87.89 (87.03 to 88.11)	3.9099	0.5873
≥8	38.88 (37.73 to 40.06)	92.20 (91.75 to 92.62)	4.9819	0.6629
≥9	29.84 (28.76 to 30.94)	95.34 (94.99 to 95.68)	6.4089	0.7359
≥10	22.11 (21.14 to 23.12)	97.43 (97.16 to 97.68)	8.6053	0.7994
≥11	16.36 (15.49 to 17.26)	98.56 (98.35 to 98.75)	11.3733	0.8486
≥12	12.03 (11.27 to 12.83)	99.28 (99.12 to 99.41)	16.6441	0.8861
≥13	8.33 (7.69 to 9.01)	99.60 (99.48 to 99.69)	20.7021	0.9204
≥14	5.49 (4.97 to 6.07)	99.79 (99.70 to 99.85)	25.9892	0.9471
≥15	3.25 (2.84 to 3.70)	99.85 (99.77 to 99.90)	21.6398	0.9690
≥16	2.14 (1.82 to 2.52)	99.90 (99.83 to 99.94)	20.9674	0.9796
≥17	1.12 (0.89 to 1.40)	99.97 (99.92 to 99.99)	32.7436	0.9867
≥18	0.65 (0.48 to 0.88)	100 (99.97 to 100)	-	0.9935
≥19	0.34 (0.22 to 0.52)	100 (99.97 to 100)	-	0.9966
≥20	0.12 (0.06 to 0.24)	100 (99.97 to 100)	-	0.9988
≥21	0.04 (0.01 to 0.14)	100 (99.97 to 100)	-	0.9996
≥22	0.02 (0.00 to 0.10)	100 (99.97 to 100)	-	0.9996
≥23	0	100	-	1.0000

For peer review only

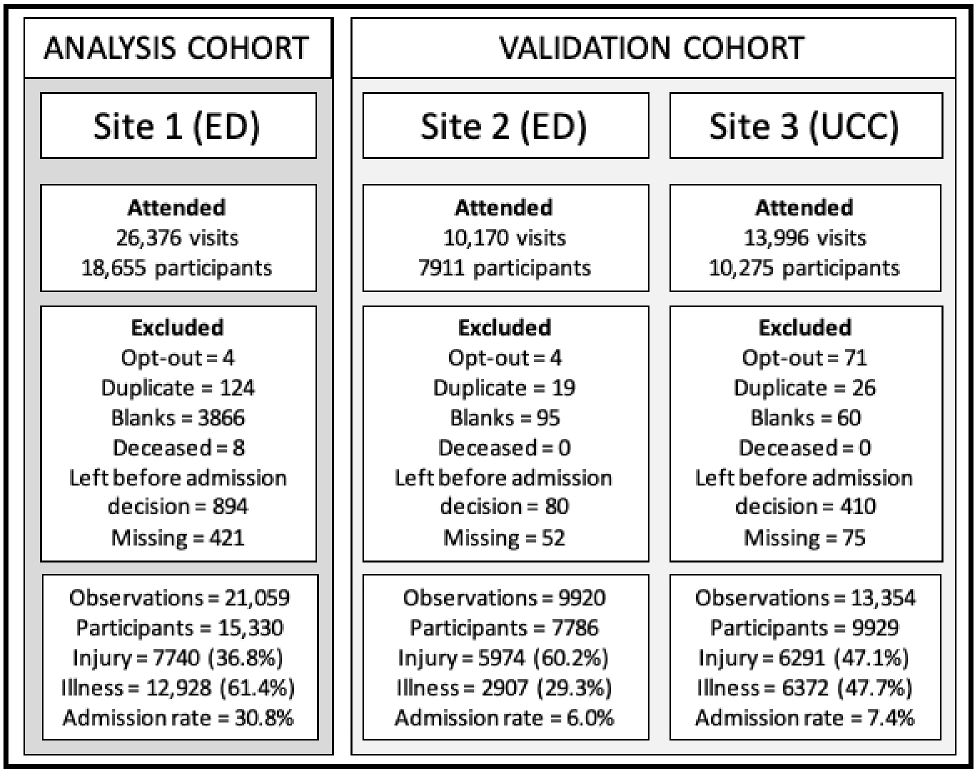

Figure 1 - Development of the dataset

211x168mm (72 x 72 DPI)

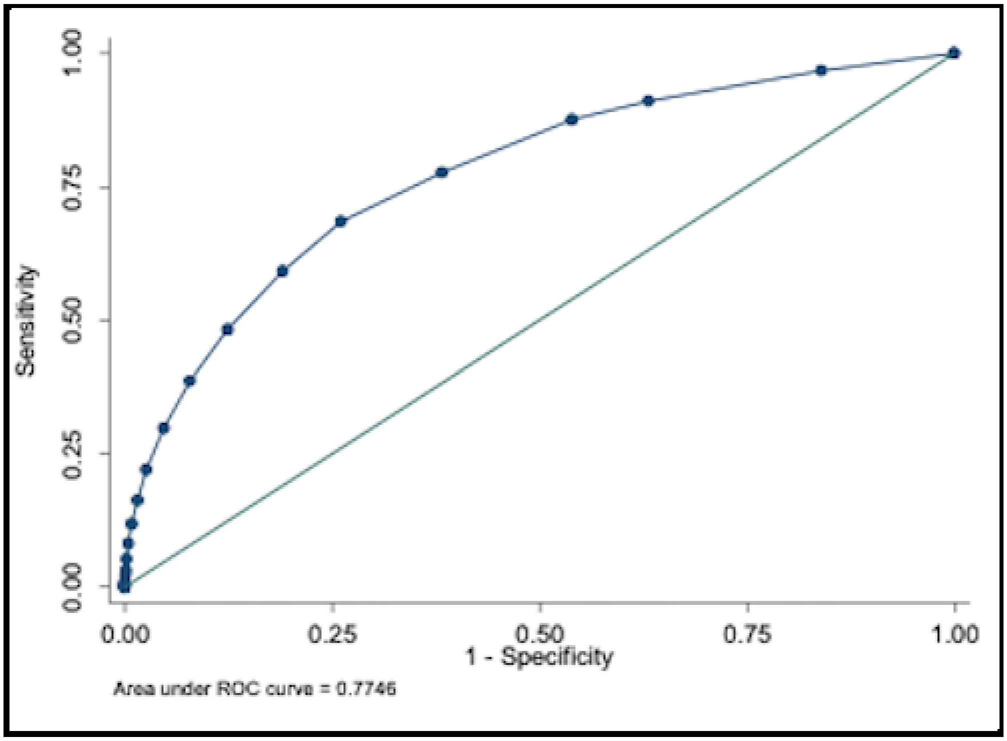

Figure 2 Area under the ROC curve of the PAGE scoring tool

Statistical Appendix

This appendix contains only details not included in the main paper. It is intended that this Appendix will be made available online-only as a supplementary file.

Use of variables in the model

All of the variables were used in the model with the form of the individual variables and their suitability for inclusion in the model considered and adjusted as necessary.

Categorical variables with small categories

The category of behaviour 'inappropriate' was recorded for 54 children (<1%). This was too small a category to include in the modelling, and it was not clear what other category to amalgamate it with, as both 'floppy' and 'listless' could be considered 'inappropriate'. These were marked as missing, removing those children from the analysis. The four categories of abnormal breathing (audible grunt, wheeze, stridor and tracheal tug) were very small (0 to 3%) and so breathing was re-categorised as 'normal' and 'abnormal'. The category 'severe recession' was recorded for only 311 children over the three sites (between 0 and 1% per site), so 'severe' and 'moderate' recession were combined.

Continuous variables

During the regression modelling the fractional polynomial command made suggestions for the transformation of continuous variables. Age was included as reciprocal fractional polynomial of the original variable. Temperature and respiratory

For peer review only - http://bmjopen.bmj.com/site/about/guidelines.xhtml

rate had a linear relationship with admittance and were included in the model as continuous variables.

For heart rate and oxygen saturation the plots of the fractional polynomials laid over the data were examined to visually assess how the models fit. These plots and the findings from the exploratory analysis jointly led decisions on the ultimate form of each of the variables.

- **Heart rate:** The fractional polynomial suggested a linear association with admittance. Examining the plot revealed that although a linear relationship is broadly appropriate it does a poor job of capturing the two large dips in probability of admittance. A more complex plot would have captured better the association between heart rate and admittance. With the large sample size such a curve could have been fitted without being overly concerned about overfitting. However, this would have defeated the overall aim of an easily used paper-based point scoring tool by requiring multiple coefficients in the final model. Heart rate was categorised as under 75, 75 to 125 and over 125.
- **Oxygen saturation:** The multivariable fractional polynomial modelling suggested that the raw oxygen saturation variable taken to the power of -2 was the best fitting form. On examination of the fractional polynomial plot it was clear that the plot fitted the data very well for oxygen saturations up to 90%, but above 90%, where 99% of the data lay, the polynomial failed to capture the more erratic association between saturation and admittance. Accordingly, a decision was taken to categorise the oxygen saturation variable based on the frequencies of the responses at each saturation

For peer review only - http://bmjopen.bmj.com/site/about/guidelines.xhtml

percentage, its observed relationship with admittance from the graph and on the previous PAT-POPS model (which aligns with clinical opinion of oxygen saturation cut-offs): 95%-100%; 90%-94%; <90%.

Being aware of the statistical implications of categorising continuous variables these decisions were not made lightly, however it was clear that no fractional polynomial would be able to capture the nature of the relationship better than categorisation.

Internal validation

Figure A shows the internal validation calibration plot. The blue ‘lowess’ line fitting the reference line so well is a demonstration of how well the model is fitting the data. Given the large sample size however this is to be expected. **Table A** shows the various internal validation results. The ‘apparent’ results are the average measures of validation for the 500 models created from the 500 bootstraps of the data. The ‘test’ results show the average performance of the models developed for each of those 500 bootstraps when applied to the original dataset. The ‘optimism’ results are the ‘apparent’ results subtracted from the ‘test’ results. ‘test’ results similar to ‘apparent’ results (and subsequently small ‘optimism’ results) suggest strong internal validity.

For peer review only

Figure A. Calibration plot for internal validation.

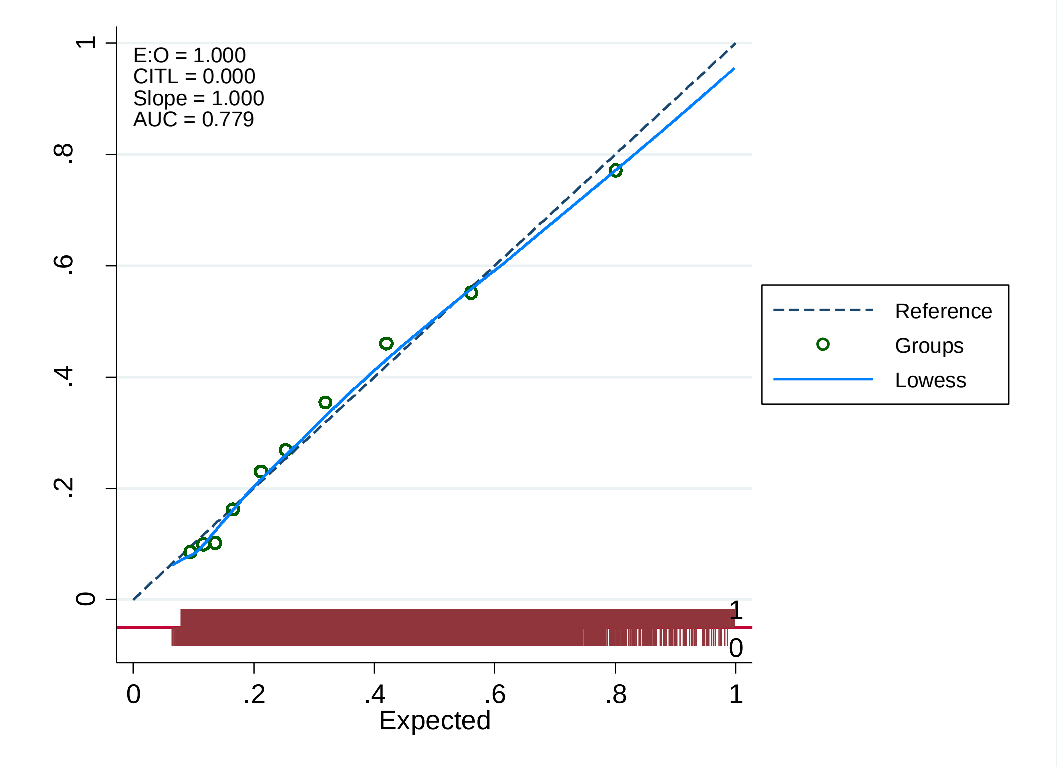

Table A. Results of internal validation.

Measure	Bootstraps	Mean	Standard deviation	Minimum	Maximum
**Apparent C-index**	500	.7790287	.0035788	.7685792	.7889659
**Apparent CITL**	500	0.00000000393	0.0000000181	-0.000000103	0.000000190
**Apparent slope**	500	1	0.0000000549	1	1.000001
**Test C-index**	500	.7780273	.0005261	.7751072	.7791697
**Test CITL**	500	.0000289	.0158041	-.0458299	.0523462
**Test slope**	500	.9934335	.0175813	.9502897	1.043822
**Optimism C-index**	500	.0010013	.0035684	-.0097837	.0107504
**Optimism CITL**	500	-.0000289	.0158041	-.0523462	.0458299
**Optimism slope**	500	.0065665	.0175813	-.0438216	.0497103

For peer review only - http://bmjopen.bmj.com/site/about/guidelines.xhtml

External validation: Site 2

Model reproducibility or transportability

The C-index for this membership model was 0.8078. The closer this number is to 1 the more likely it is that this represents model transportability rather than model reproducibility. So, in carrying out external validation using the Site 2 dataset an assessment is being made of whether the model works well in a different but related population. This was as expected given that from the exploratory analysis it could be seen that Site 2 represent a different mix of ethnicities as well as generally older and healthier patients.

Calibration and discrimination

The Brier score was lower in the Site 2 data (0.0653) than in the model development dataset, suggesting very good prediction accuracy in this external dataset. At 0.9864908 (0.896986 to 1.075996) the calibration slope was also close to the ideal of 1. Calibration-in-the-large (CITL) was less impressive at -1.218977 (-1.305886 to -1.132068). This suggests that the predicted probabilities were higher than the observed proportions. So, children in Site 2 were less likely to be admitted than the model predicted; a child with equivalent characteristics presenting at Site 1, for example, would be more likely to be admitted. This can be seen in the calibration plot **(Figure B)** where the observed probabilities are consistently lower than the expected probabilities, especially as the expected probabilities near 1. This was also evident in the E/O ratio of 2.7. This is the expected/observed ratio, how many were expected to be admitted based on the model versus how many actually were

For peer review only - http://bmjopen.bmj.com/site/about/guidelines.xhtml

admitted, so ideally this would be 1. That it is higher here suggests less people were admitted than would have been expected from the model. In terms of discrimination, the C-index was 0.7626 (0.74244 to 0.78279) **(Figure B)**.

Figure B. Calibration plot for Site 2 external validation.

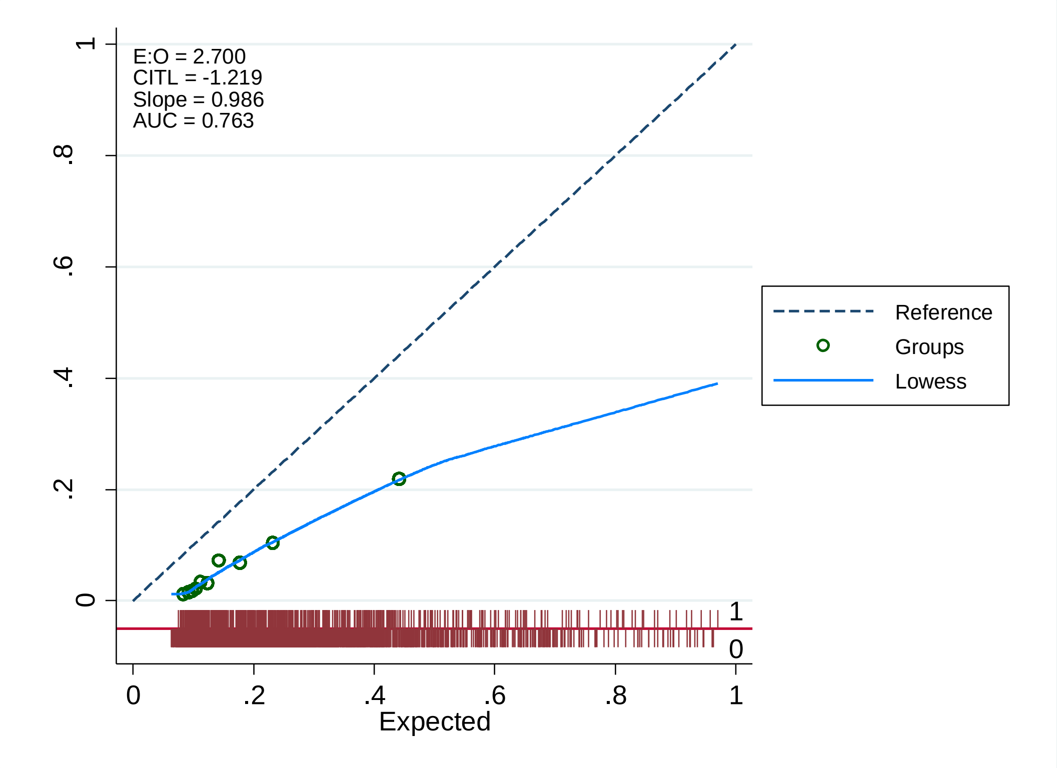

only

External validation: Site 3

Model reproducibility of transportability

The C-index for this membership model was 0.8154. This suggests that Site 1 and Site 3 are also quite different from each other in terms of their populations and therefore model transportability was assessed with the following results. That these two sites differ is expected; aside from the difference in ethnic make-up of the populations, Site 1 is an ED whereas Site 3 is an Urgent care centre and therefore sees healthier children.

For peer review only - http://bmjopen.bmj.com/site/about/guidelines.xhtml

Calibration and discrimination

As with Site 2, when the model was applied to Site 3 the Brier score was a very low 0.0732. The calibration slope was over 1 at 1.075, this time suggesting overfitting, but as with Site 2 the CI also crossed 1 (0.976 to 1.123). The CITL was -1.055 (-1.123 to -0.987) suggesting predicted probabilities were higher than the observed proportions, again evident in **Figure C**. The E/O ratio was 2.3. The C-index was 0.7533 (0.734 to 0.770).

Figure C. Calibration plot for Site 3 external validation.

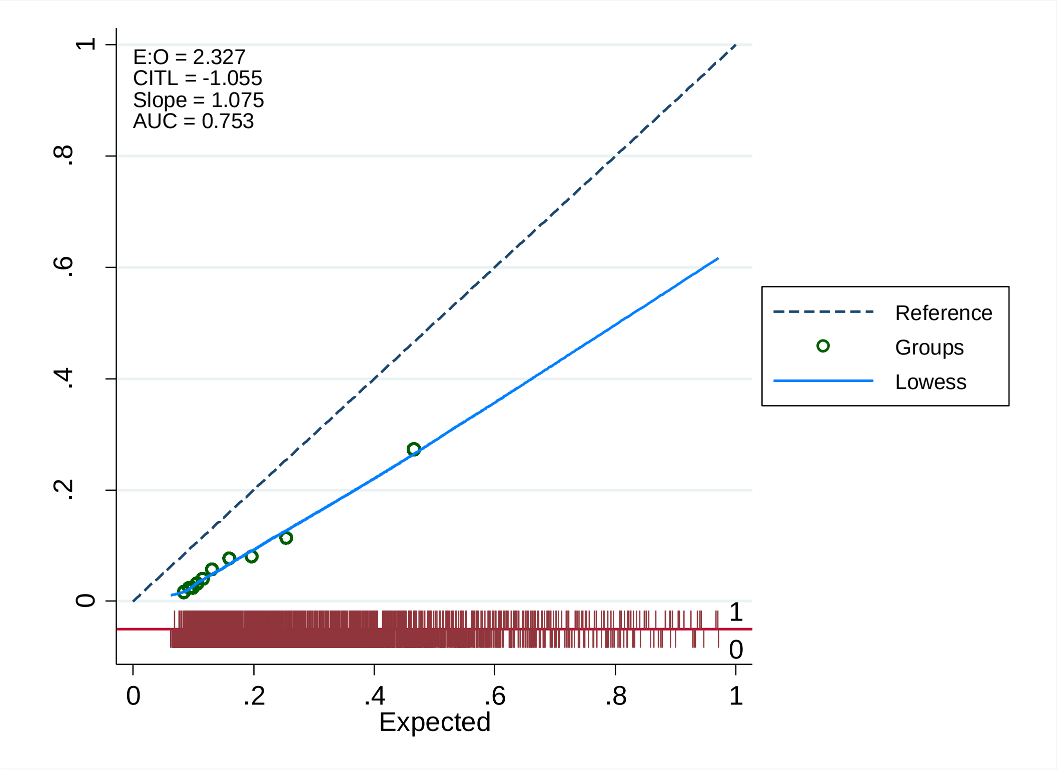

Overall

From these results it can be determined that the model transportability is good overall. With only slight over/under-fitting present at each of the external validation sites and C-indexes only modestly smaller than was found in the original model. It is

For peer review only - http://bmjopen.bmj.com/site/about/guidelines.xhtml

clear however that the model may over-estimate the probability of admission in external populations, probably owing to the admission rate at the development site (Site 1).

For peer review only

For peer review only - http://bmjopen.bmj.com/site/about/guidelines.xhtml

TRIPOD Checklist: Prediction Model Development

Section/Topic	Item	Checklist Item	Page
**Title and abstract**			
Title	1	Identify the study as developing and/or validating a multivariable prediction model, the target population, and the outcome to be predicted.	1
Abstract	2	Provide a summary of objectives, study design, setting, participants, sample size, predictors, outcome, statistical analysis, results, and conclusions.	3-4
**Introduction**			
Background and objectives	3a	Explain the medical context (including whether diagnostic or prognostic) and rationale for developing or validating the multivariable prediction model, including references to existing models.	5-6
	3b	Specify the objectives, including whether the study describes the development or validation of the model or both.	6
**Methods**			
Source of data	4a	Describe the study design or source of data (e.g., randomized trial, cohort, or registry data), separately for the development and validation data sets, if applicable.	6-7
	4b	Specify the key study dates, including start of accrual; end of accrual; and, if applicable, end of follow-up.	6
Participants	5a	Specify key elements of the study setting (e.g., primary care, secondary care, general population) including number and location of centres.	6-7
	5b	Describe eligibility criteria for participants.	6-7
	5c	Give details of treatments received, if relevant.	–
Outcome	6a	Clearly define the outcome that is predicted by the prediction model, including how and when assessed.	7
	6b	Report any actions to blind assessment of the outcome to be predicted.	–
Predictors	7a	Clearly define all predictors used in developing or validating the multivariable prediction model, including how and when they were measured.	7
	7b	Report any actions to blind assessment of predictors for the outcome and other predictors.	–
Sample size	8	Explain how the study size was arrived at.	8
Missing data	9	Describe how missing data were handled (e.g., complete-case analysis, single imputation, multiple imputation) with details of any imputation method.	13-14
Statistical analysis methods	10a	Describe how predictors were handled in the analyses.	9
	10b	Specify type of model, all model-building procedures (including any predictor selection), and method for internal validation.	9-10
	10d	Specify all measures used to assess model performance and, if relevant, to compare multiple models.	10-11
Risk groups	11	Provide details on how risk groups were created, if done.	–
**Results**			
Participants	13a	Describe the flow of participants through the study, including the number of participants with and without the outcome and, if applicable, a summary of the follow-up time. A diagram may be helpful.	Fig 1
	13b	Describe the characteristics of the participants (basic demographics, clinical features, available predictors), including the number of participants with missing data for predictors and outcome.	Table 1
Model development	14a	Specify the number of participants and outcome events in each analysis.	Fig 1
	14b	If done, report the unadjusted association between each candidate predictor and outcome.	–
Model specification	15a	Present the full prediction model to allow predictions for individuals (i.e., all regression coefficients, and model intercept or baseline survival at a given time point).	Table 2
	15b	Explain how to the use the prediction model.	17
Model performance	16	Report performance measures (with CIs) for the prediction model.	15-16
**Discussion**			
Limitations	18	Discuss any limitations of the study (such as nonrepresentative sample, few events per predictor, missing data).	19
Interpretation	19b	Give an overall interpretation of the results, considering objectives, limitations, and results from similar studies, and other relevant evidence.	17-19
Implications	20	Discuss the potential clinical use of the model and implications for future research.	20
**Other information**			
Supplementary information	21	Provide information about the availability of supplementary resources, such as study protocol, Web calculator, and data sets.	4,15
Funding	22	Give the source of funding and the role of the funders for the present study.	~~1~~ 2

We recommend using the TRIPOD Checklist in conjunction with the TRIPOD Explanation and Elaboration document.

BMJ Open

An observational cohort study with internal and external validation of a predictive tool for identification of children in need of hospital admission from the emergency department: the Paediatric Admission Guidance in the Emergency department (PAGE) score

Journal:	*BMJ Open*
Manuscript ID	bmjopen-2020-043864.R1
Article Type:	Original research
Date Submitted by the Author:	24-Nov-2020
Complete List of Authors:	Rowland, Andrew; University of Salford<br>Cotterill, Sarah; The University of Manchester, Centre for Biostatistics<br>Heal, Calvin; The University of Manchester, Manchester Academic Health Science Centre; Salford Royal NHS Foundation Trust<br>Garratt, Natalie; Salford Royal Hospital<br>Long, Tony; University of Salford<br>Bonnett, Laura; University of Liverpool, Biostatistics<br>Brown, Stephen; Salford Royal Hospital<br>Woby, Steve ; Salford Royal NHS Foundation Trust,<br>Roland, Damian; University of Leicester, Health Sciences; University Hospitals of Leicester NHS Trust, Children's Emergency Department
**Primary Subject Heading**:	Emergency medicine
Secondary Subject Heading:	Paediatrics
Keywords:	Paediatric A&E and ambulatory care < PAEDIATRICS, Protocols & guidelines < HEALTH SERVICES ADMINISTRATION & MANAGEMENT, ACCIDENT & EMERGENCY MEDICINE

SCHOLARONE™
Manuscripts

I, the Submitting Author has the right to grant and does grant on behalf of all authors of the Work (as defined in the below author licence), an exclusive licence and/or a non-exclusive licence for contributions from authors who are: i) UK Crown employees; ii) where BMJ has agreed a CC-BY licence shall apply, and/or iii) in accordance with the terms applicable for US Federal Government officers or employees acting as part of their official duties; on a worldwide, perpetual, irrevocable, royalty-free basis to BMJ Publishing Group Ltd ("BMJ") its licensees and where the relevant Journal is co-owned by BMJ to the co-owners of the Journal, to publish the Work in this journal and any other BMJ products and to exploit all rights, as set out in our licence.

The Submitting Author accepts and understands that any supply made under these terms is made by BMJ to the Submitting Author unless you are acting as an employee on behalf of your employer or a postgraduate student of an affiliated institution which is paying any applicable article publishing charge ("APC") for Open Access articles. Where the Submitting Author wishes to make the Work available on an Open Access basis (and intends to pay the relevant APC), the terms of reuse of such Open Access shall be governed by a Creative Commons licence – details of these licences and which Creative Commons licence will apply to this Work are set out in our licence referred to above.

Other than as permitted in any relevant BMJ Author's Self Archiving Policies, I confirm this Work has not been accepted for publication elsewhere, is not being considered for publication elsewhere and does not duplicate material already published. I confirm all authors consent to publication of this Work and authorise the granting of this licence.

For peer review only

An observational cohort study with internal and external validation of a predictive tool for identification of children in need of hospital admission from the emergency department: the Paediatric Admission Guidance in the Emergency department (PAGE) score

Rowland AG[1,2], Cotterill S[3], Heal C[3], Garratt N[4], Long T[1], Bonnett LJ[5], Brown S[4], Woby S, [1,4] and Roland D[6,7]

1 CYP@Salford, School of Health and Society, the University of Salford, Salford, UK

2 Emergency Department, North Manchester General Hospital, The Pennine Acute Hospitals NHS Trust, Manchester, UK

3 Centre for Biostatistics, School of Health Sciences, the University of Manchester, Manchester, UK

4 The Northern Care Alliance NHS Group, UK

5 Department of Biostatistics, University of Liverpool, UK

6 SAPHIRE Group, Health Sciences, University of Leicester, Leicester, UK

7 Paediatric Emergency Medicine Leicester Academic (PEMLA) Group, Children's Emergency Department, Leicester Royal Infirmary, Leicester, UK

Correspondence to

Dr. Damian Roland, Consultant in Children's Emergency Medicine, Children's Emergency Department, Leicester Royal Infirmary, Leicester, LE1 5WW

dr98@le.ac.uk

+44 (0)7727158213

For peer review only

Conflicts of Interest

None of the authors has anything to declare.

Contributions

Initial study design was by DR, AR, TL, SC, CH and SW with NG, LB and SB contributing to development. Study data was collected, processed and evaluated by SB, CH and SC. LB contributed to statistical analysis and review. NG managed the project under the leadership of DR and AR. All authors contributed to both initial and final drafts and agreed to the submission of the final draft.

Funding

This paper presents independent research funded by the National Institute for Health Research (NIHR) under its Research for Patient Benefit (RfPB) Programme (Grant Reference Number PB-PG-0815-20034). The views expressed are those of the authors and not necessarily those of the NIHR or the Department of Health and Social Care.

For peer review only

ABSTRACT

Objectives: To devise an assessment tool to aid discharge and admission decision-making in relation to children and young people in hospital urgent and emergency care facilities, and thereby improve the quality of care that patients receive, using a clinical prediction modelling approach

Design: Observational cohort study with internal and external validation of a predictive tool

Setting: Two general emergency departments and an urgent care centre in the North of England.

Participants: The eligibility criteria were children and young people 0-16 years of age who attended one of the three hospital sites within one NHS organisation. Children were excluded if they opted out of the study, were brought to the emergency department (ED) following their death in the community, or arrived in cardiac arrest when the heart rate and respiratory rate would be unmeasurable.

Main outcome measures: Admission or discharge. A participant was defined as being admitted to hospital if they left the ED to enter the hospital for further assessment, (including being admitted to an observation and assessment unit or hospital ward), either on first presentation or with the same complaint within seven days. Those who were not admitted were defined as having been discharged.

Results: The study collected data on 36365 participants. 15328 participants were included in the final analysis cohort (21045 observations) and 17710 participants were included in the validation cohort (23262 observations). There were 14 variables entered into the regression analysis. Of the 13 that remained in the final model, 10 were present in all 500 bootstraps. The resulting **P**aediatric **A**dmission **G**uidance in

For peer review only

the **E**mergency Department (PAGE) score demonstrated good internal validity. The C-index (AUROC) was 0.779 (95% CI 0.772 to 0.786).

Conclusions: For units without the immediate availability of paediatricians the PAGE score can assist staff to determine risk of admission. Cut off values will need to be adjusted to local circumstance.

Study protocol: The study protocol has been published in an open access journal:

Riaz, S., Rowland, A., Woby, S. et al. Refining and testing the diagnostic accuracy of an assessment tool (PAT-POPS) to predict admission and discharge of children and young people who attend an emergency department: protocol for an observational study. BMC Pediatr 18, 303 (2018). https://doi.org/10.1186/s12887-018-1268-7

Trial registration: The protocol has been published and the study registered (NIHR RfPB Grant: PB-PG-0815-20034; ClinicalTrials.gov: 213469)

Strengths and limitations of this study

- This study was novel in that it was based on data from children attending three non-tertiary (non-specialist) hospitals (n=36,365). Non specialist hospitals are where the majority of children are seen by emergency and urgent care practitioners in the NHS in England
- The Paediatric Admission Guidance in the Emergency department (PAGE) score, was built from the clinical prediction model derived by both an internal and external data validation set.
- Admission rates varied between hospitals and while internal and external validation occurred the impact of prospective use to guide decision will need confirmation.

For peer review only

INTRODUCTION

Attendances at emergency departments (ED) by children and young people have risen year on year in the United Kingdom and are now in excess of 4 million per year **[1]**. The majority of these attendances are at services where the professional contact is by a practitioner without formal paediatric training. Given that a large proportion of these children and young people are discharged safely, the delivery of emergency and acute care does not rely on direct paediatric review.

However, systems are needed to recognise children who require further observation and investigation. Triage processes are well-established to determine the order in which patients should receive clinical review. Increasingly, a fuller assessment, including the patient's vital signs, is used to determine an acuity score to define risk of illness or deterioration. These scores, often termed early warning scores (EWS), originated in adult in-patient environments where it was recognised that death or intensive care admission was often preceded by derangement of the patient's physiology **[2]**. These findings led to the development of paediatric early warning scores (PEWS) which have been shown to have utility in the detection of deterioration, but not always in the prevention of mortality **[3]**. The face validity of such scores led to widespread utilisation and attempts to employ them in different emergency and pre-hospital environments from those where the scores were devised. Initial reviews demonstrated relatively poor performance **[4]**, in turn leading to the development of bespoke scores validated in emergency departments themselves.

For peer review only - http://bmjopen.bmj.com/site/about/guidelines.xhtml

There is no existing gold standard outcome measure for the decision to admit or discharge a child or young person from the ED **[5]**, and the decision to admit is a complex one, which can vary between clinicians and hospitals. One early warning scoring system, the Paediatric Observation Priority Score (POPS), recommended by the Intercollegiate Committee for Standards for Children and Young People in Emergency Care Settings **[6]** has shown initial promise in aiding recognition of unwell children but also aiding safe discharge decisions **[7,8]**.

A modified version of POPS, developed at The Pennine Acute Hospitals NHS Trust (PAT-POPS), has been shown to have greater discriminatory power than an inpatient PEWS **[9]**. The majority of EWS systems are derived and validated at large teaching institutions ("tertiary centres") and so may not be applicable outside tertiary centres **[10]**. In this study PAT-POPS was used as the initial starting point to derive and validate a score to aid disposition decisions in non-tertiary centres. This would have external validity in the majority of locations where children and young people are assessed in the United Kingdom and potentially beyond.

METHODS

A published protocol is available **[11]** and only the core components of the methods are summarised in this paper. In this paper "participant" means a patient recruited into the study at one of the study sites.

Patient And Public Involvement

The study was supported by a patient advisory group which provided input to the programme of research. This patient advisory group met with author TL during the

For peer review only - http://bmjopen.bmj.com/site/about/guidelines.xhtml

study. Patients partnered with the study team for the design of the study, the informational material to support the opt-out consent process. At the end of the study twenty-one parents were consulted in one of the EDs on how the cut point might be made. Most found this a particularly difficult task, hovering around the centre of the scale. When the notion was introduced of a supporting community service that would know about their child and for which they had a contact number if worried, a remarkable difference was observed. All but two (both fathers) considered correct discharge to be more important than correct admission, and, overall, most thought that about 60-70% correct discharge was optimal. Two mothers would be sufficiently confident for a much higher specificity, being prepared to discharge 90-95% patients correctly.

Sample Population

The study population was recruited consecutively. Data collection was prospective over a whole year (1 March 2018 to 28 February 2019) to avoid the effects of bias from seasonal variability. The eligibility criteria were children and young people 0-16 years of age who attended one of three hospital sites within one NHS Trust (NHS organisation) in Greater Manchester, UK. Children were excluded if they opted out of the study, were brought to the ED following their death in the community, or arrived in cardiac arrest when the heart rate and respiratory rate would be unmeasurable.

Admission

A participant was defined as being admitted to hospital if they left the ED to enter the hospital for further assessment, (including observation and assessment unit or hospital ward), either on first presentation or with the same complaint within seven

For peer review only - http://bmjopen.bmj.com/site/about/guidelines.xhtml

days. This was approved by the study patient and public involvement group, which saw admission and discharge in such terms, and by discussion with three ED doctors.

The decision to admit the participant was made by a clinician (either a doctor or a nurse practitioner). They followed existing guidelines, using usual methods of clinical judgement, and were blinded to the score as this had not yet been derived at the point of participant assessment (but had access to all vital signs and observations normally collected). Admission data from all three hospitals was accessed from existing NHS trust electronic systems.

Predictors

All of the variables in the modified PAT-POPS plus additional variables included in other scores were considered for inclusion in the PAGE score (**Table 1**). Other data collection included reason for attendance at the ED, diagnosis, death in the ED, children leaving the ED before admission decision, children's characteristics (age, gender, and ethnicity), investigated deaths and serious incidents. These variables were chosen as in the authors clinical experience they may have dependency on the decision to admit but have also not be formally studied in previous analyses **[4]**.

[INSERT TABLE 1 HERE]

Sample Size

In the protocol it was estimated that 9000 children were needed for the development of the prediction model and 7000 children in the independent validation. The minimum sample size needed to do both analyses was therefore estimated as 16000

For peer review only - http://bmjopen.bmj.com/site/about/guidelines.xhtml

children. More data than needed was allowed for (and this approach was granted at ethics review) due to the need to collect data for a full year to capture seasonal variation in childhood illness and injury. Intermittent data collection would not help implementation of the tool and would have required the employment of specific staff for the project, which would have been significantly more costly.

Analysis

Analysis was conducted in StataMP 14 **[12]** using two-sided 95% confidence intervals and the 5% significance level. Analysis was reported according to the TRIPOD **[13]** and STARD **[14]** reporting guidelines. A flow diagram summarises participant opt-out, recruitment and data collection, by site (**Figure 1**).

[INSERT FIGURE 1 HERE]

Final analysis was undertaken after all data had been entered into the database, and the database had been cleaned and locked. Children were excluded from the analysis if the outcome variable (admission) was missing or if all the independent variables were missing. For variables with over 4% rates of missingness data was imputed using hot-deck imputation **[15]**. This involves stratifying participants by key predictive variables (injury/illness status, admission status and age) and replacing missing values with those of a participant from the same strata. Participants were described with respect to the variables in the model, both overall and by site, reported as number (%) for categorical variables; mean (standard deviation (sd), min, max) for normally distributed variables; median (inter-quartile range (IQR), min, max) for other numeric variables.

For peer review only - http://bmjopen.bmj.com/site/about/guidelines.xhtml

Stage 1: Model development

Children from one hospital site were utilised for the model development. Logistic regression models were developed with hospital admission as the outcome and including all candidate variables. Due to several of the variables being non-linearly associated with the outcome a closed test procedure was used to determine the best functional form of each continuous variable and concurrently whether it should be included. This involved starting with a model that included all potential predictors and testing the best fitting fractional polynomial form of each continuous variable individually **[16]**. Variables were removed from the model according to p-values with any less than 0.10 being excluded. For categorical variables, if at least one category was significant all were included initially. Quality of the data and risk of bias was assessed using PROBAST **[17]**.

Stage 2: Internal validation

The calibration score and calibration slope were examined to assess how well the predictions from the model matched the data and calibration plots were used to compare agreement between predicted and observed injury and illness. Discrimination was also considered, to measure how well the model separated between individuals who were admitted and those who were not (C-statistic, which is equivalent to area under the ROC curve).

The Brier score has been reported. Internal validation was performed by applying the original model to 500 bootstrapped samples. The discrimination and calibration performance of the model in each of the bootstrap samples was compared with the

For peer review only - http://bmjopen.bmj.com/site/about/guidelines.xhtml

model fitted to the original data to provide a measure of optimism. Due to the large sample size, it was not necessary to adjust for the optimism **[18]**. The inclusion/exclusion of any predictors which featured in the selected model but only rarely across the bootstrap samples (or vice versa) was noted.

The output of Stage 1 and 2 was the **P**aediatric **A**dmission **G**uidance in the **E**mergency department (PAGE) score which aimed to predict hospital admission, based on the weight of each predictor.

Stage 3: External validation

Assessing model performance

External validation was undertaken using data from two other hospitals. The developed model was applied to each external dataset, and calibration and discrimination measures were reported, as above.

Recalibration

After looking at both the development and the external datasets, and with clinical opinion, consideration was given to which dataset most closely represents the majority of UK emergency departments at which children attend, and accordingly whether or not to recalibrate the model based on one of the external validation datasets.

Stage 4: Developing a point scoring system

The model's regression coefficients were used to assign integer points to each level of each risk factor, and a reference table of risk per possible points total was

For peer review only - http://bmjopen.bmj.com/site/about/guidelines.xhtml

produced, following established guidelines **[19]**. Together these provide a clinically useful score. By applying the points scores to the development dataset it was possible to calculate the sensitivity, specificity, positive and negative likelihood ratios of PAGE (index test) in predicting admission (reference test) with 95% confidence intervals. A cross tabulation of the results of the index test by the results of the reference test was reported, including indeterminate and missing results.

Consensus Meeting

A meeting was held to examine the statistical data, and to agree which cut points of the PAGE score were most suitable to predict (i) safe admission decision and (ii) safe discharge decision, including consideration of what weight to give to sensitivity and specificity in making the decision. All of our research team, plus paediatric ED clinicians and an independent methodologist, were invited to attend.

Stage 5: Further analyses

The usefulness of the PAGE score was assessed by calculating the sensitivity, specificity, positive and negative likelihood ratios at the chosen cut-points, to predict admission and discharge.

Ethics and Research Governance

The West Midlands Research Ethics Committee approved this project (**17/WM/0436**) on the 20th December 2017. An opt-out consent design was approved, and there were no plans to disseminate the results to participants.

For peer review only - http://bmjopen.bmj.com/site/about/guidelines.xhtml

RESULTS

The unit of analysis was a visit by a child to the ED. **Figure 1** shows the numbers and reasons for exclusions of visits (and participants) from the dataset. Children were omitted because they opted out of the study (n=4, Site 1), died in the ED (n=8, Site 1) or left the ED before an admission decision was made (n=892, Site 1). Duplicate entries where participants had more than one entry with the same date and time (n=199, Site 1) were deleted.

Missing visits

There were two types of visit that were excluded completely from the development dataset due to extensive missing data. The first (marked as 'blanks' in **Figure 1**) were missing all data except for patient demographics. After data collection was completed, we discovered that Site 1 had a two-tier system which funnels off particularly 'well' children into a separate nurse-led service where no further study information was recorded (n=3865, Site 1). The second were visits where data was missing for enough clinical measures and judgements to prevent imputation and inclusion in the analysis This was likely due to them being measurements that would not routinely be taken for certain types of attendances, such as minor injuries (n=421, Site 1).

Missing variables

Most variables had very low rates of missingness (0 to 2%) and no imputation was required. Four variables: heart rate, temperature, respiratory rates and oxygen saturation had higher rates, which varied by site: Site 1 (8-11%), Site 2 (2-4%) and

For peer review only - http://bmjopen.bmj.com/site/about/guidelines.xhtml

Site 3 (1-2%). Reasons for the missingness, and differences in missingness by site, are not clear. Values were imputed using hot-deck imputation **[15]**.

Descriptive statistics / summary of population

Table 1 summarises the demographic and medical characteristics of the sample population at each site. This comprises all participants who met the inclusion criteria. The admission rate of Site 1 (the development dataset) was 32% compared to 6% at Site 2 and 8% at Site 3. Compared to the external validation sites, children in the development dataset were younger, showed signs of being more unwell (higher heart rate, more prevalence of recession and breathing abnormalities) and were more likely to show cause for concern for the nurses. Temperature, oxygen saturation, responsiveness and behaviour were very similar across the sites.

The form of variables and their suitability for inclusion in the model were considered and adjusted as necessary. Categorical variables were re-coded to avoid any small categories. Abnormal breathing was re-categorised as ‘normal’ and ‘abnormal’. ‘Severe’ and ‘moderate’ recession were amalgamated. Two continuous variables (heart rate and oxygen saturation) were categorised as in their continuous form we were unable to model them adequately whilst keeping the final score readily usable in clinical practice.

Regression results

The final multivariable logistic regression model is shown in **Table 2**.

Responsiveness was omitted from the final model, because once the other variables were accounted for, it had no effect on admission. All other variables were retained

For peer review only - http://bmjopen.bmj.com/site/about/guidelines.xhtml

For peer review only

in the final model and had a statistically significant relationship with admission except for the ‘under 75 bpm’ heart rate category.

[INSERT TABLE 2 HERE]

The younger the child the more likely admission is and the effect is much stronger for much younger participants. Compared to those aged 4 to 16 years old, those aged 0-1 months had an increase in admission odds of 1.880 (1.876 to 1.885). Low oxygen saturation was the strongest predictor of admission in the study and Nurse judgement was strongly associated with admission. Variables that were introduced in this study as additional to the existing PAT-POPS score were multi-morbidity, (1.94 (1.60 to 2.35)), arrival by ambulance (2.42 (2.22 to 2.63)) and being advised to attend by a medical professional (2.16 (1.97 to 2.36)).

Internal Validation

As would be expected from such a large sample size, the model demonstrated good internal validity. The discrimination (C-index) of the model in the original dataset was 0.779 (95% CI 0.772 to 0.786) and the mean in the bootstrap samples was 0.779 (95% CI 0.767 to 0.789) suggesting no model optimism. Full calibration and discrimination results are reported in the supplementary file.

Calibration

The Brier score was 0.1664, the calibration slope was 1.000 and calibration-in-the-large was -0.000. The calibration plot indicates that predictions from the model

For peer review only - http://bmjopen.bmj.com/site/about/guidelines.xhtml

For peer review only

match the data very well for low to moderate risk individuals. Above this, the model slightly over-predicts the probability of admission.

External validation

The external validation was undertaken in each of the two external datasets separately (**Table 3**).

[INSERT TABLE 3 HERE]

These results indicate that the model transportability is good overall, with only slight over/under-fitting present at each of the external validation sites and C-indexes (0.763 and 753 for site 2 and 3 respectively), only modestly smaller than were found in the original model. It is clear however that the model may over-estimate the probability of admission in external populations, probably owing to the unusually high admission rate of the development site (site 1).

Of the three sites, site 1 is most typical of United Kingdom EDs in respect of annual attendances and size of unit. This being the case, recalibration of the model so it performed better in the two external datasets was not necessary. The original model was carried forward to the stage of developing the point scoring system.

Derivation of Paediatric Admission Guidance in the Emergency department (PAGE) score

The PAGE score has a minimum value of 0 and a maximum of 30 (Table 4). The characteristic that yields the largest numbers of points is having a lower than normal

For peer review only - http://bmjopen.bmj.com/site/about/guidelines.xhtml

oxygen saturation, worth 4 or 5 points depending on severity. Age, against clinical expectation, seemed only predictive of admission when the participant was younger than 6 months.

[INSERT TABLE 4 HERE]

The sensitivity and specificity of each potential cut-point of the tool is shown in **table 5**. As the development of the point-scoring system was effectively a simplification of the regression model, we expected a relative decrease in its predictive power. The difference in the discrimination of the regression model and the resulting point scoring tool however proved to be minimal (**Figure 2**): a drop from 0.779 (95% CI 0.772 to 785) to 0.775 (95% CI 0.768 to 0.781).

[INSERT TABLE 5 HERE]

[INSERT FIGURE 2 HERE]

The cut-point for PAGE that most closely aligns with the sensitivity and specificity of the chosen original PAT-POPS cut-off is a score of 7 or more. This yields a sensitivity of 48.21% and specificity of 87.61%. Based on the preferences of those at the consensus meeting the chosen cut-off for their organisations would be either 6 or 7 points on the basis that consensus meeting attendees indicated a preference for giving more weight to higher specificity (ensuring the right children were discharged) than sensitivity. It is, of course, a matter for health care organisations to determine

For peer review only

For peer review only - http://bmjopen.bmj.com/site/about/guidelines.xhtml

their own cut-off based on local service arrangements, including what community services may be available to those patients who are discharged.

DISCUSSION

In this study of over 30000 children the **P**aediatric **A**dmission **G**uidance in the **E**mergency Department (PAGE) score was derived. This is the first study of its kind in non-tertiary emergency departments and therefore should have good external validity in the majority of locations in which children are treated and in which paediatricians are not immediately available. While the development site (site 1), a type 1 emergency department with trauma unit status, had a high admission rate, it is noted that the national average is 13.8% (confidence intervals unavailable) **[20]**. At site 1 the participants presenting with minor injury are streamed into an emergency nurse practitioner (ENP) stream which artificially increases the apparent admission rate. When tested on the sites with admission rates of 6% and 8% the score still performed well, highlighting that the score may have utility in organisations with a range of admission rates. The area under the ROC curve 0.779 (95% CI 0.772 to 0.785) performing better than any other previously published similar tool **[21].**

The PAGE score should not be considered a typical early warning score as it has been derived for the sole purpose of determining risk of admission, rather than risk of deterioration. A number of the components are temporally fixed (i.e. arrival by ambulance and referred by health care professional) and will not change, so the PAGE score is not a sequential scoring tool, either. However, as all the components are commonly utilised it provides an easy to measure score at initial assessment that can guide the emergency pathway utilisation for the child or young person. It is

For peer review only - http://bmjopen.bmj.com/site/about/guidelines.xhtml

important to note that age is a component of the score itself (i.e. there are not separate scores for each age group). For those used to traditional EWS approaches this may cause uncertainty as the heart rate cut off appears low for many young infants (>120 gives a score of 1 across all ages). However, it is important to remember that this is a population-derived tool and therefore at a patient level some features may not seem clinically relevant. This is why, importantly, it will be necessary for local departments to derive their own cut points for the overall PAGE score as where they are set depends on what the local services can offer both in the hospital and in the community. We also suggest in future validation it will be possible to asses it's feasibility as part of an electronic health record which will reduce the burden on health care staff calculating the total score.

The derivation of score revealed some interesting results in relation to the relative importance of the different components. While nurse judgement was the categorical variable with the strongest association with admission this was only marginal. The confidence interval for 'floppy' means that this may also be a strong predictor, but there were insufficient numbers of participants presenting with this characteristic to be able to demonstrate this. Although from a continuous variable, having an oxygen saturation below 90 was a much stronger predictor of admittance, which is in keeping with known clinical practice. However, in a group of objective measures it is perhaps surprising one of the most subjective, judgement, was one of the strongest associations. This may reflect the important predictive information that cannot or will not be obtained through clinical measurements and is clearly an area worthy of further study.

For peer review only - http://bmjopen.bmj.com/site/about/guidelines.xhtml

The PAGE score did not assess individual illness categories (it was not designed to highlight risk of sepsis) nor did it determine length of stay. Both of these can be determined in future prospective observational cohorts.

LIMITATIONS

There are limitations to the study design. Admitting or discharging a participant based on the PAGE score is only as appropriate as the original judgement of the clinicians on which the score is based. That is to say we do not know what proportion of admission decisions ultimately prove beneficial to the participant. This is not a limitation specific to our study but a wider consideration for any score that predicts admission. A number of factors influence the decision to admit a participant, and while we have attempted to determine all relevant components there may be socio-cultural factors which were missed in the initial derivation work.

CONCLUSION

For units without the immediate availability of paediatricians the PAGE score can assist staff to determine risk of admission. Cut off values will need to be adjusted to local circumstances.

REFERENCES

1. NHS Digital. https://digital.nhs.uk/data-and-information/publications/statistical/hospital-accident--emergency-activity/2018-19 Website last accessed 28th March 2020
2. NICE. Acutely Ill Patients in Hospital: Recognition and Response to Acute Illness in Adults in Hospitals, 2007
3. Roland D. Beyond Mortality: Improving outcomes for children who deteriorate in inpatient settings. J. Hosp. Med. 2019 August;14(8):512-513

For peer review only - http://bmjopen.bmj.com/site/about/guidelines.xhtml

4. Seiger N, Maconochie I, Oostenbrink R,Moll HA. Validity of different pediatric early warning scores in the emergency department Pediatrics 2013; 132
5. Lillitos PJ, Lyttle MD, Roland D On behalf of PERUKI and GAPRUKI, et al Defining significant childhood illness and injury in the Emergency Department: a consensus of UK and Ireland expert opinion Emergency Medicine Journal 2018;35:685-690
6. RCPCH. Standards for children and young people in emergency care settings 2018
7. Roland D, Lewis G, Fielding P, Hakim C, Watts A and Davies F. The Paediatric Observation Priority Score: A System to Aid Detection of Serious Illness and Assist in Safe Discharge Open Journal of Emergency Medicine 2016 4(2) 38-44
8. Roland D, Arshad F, Coats T, and Davies F. Baseline Characteristics of the Paediatric Observation Priority Score in Emergency Departments outside Its Centre of Derivation BioMed Research International, vol. 2017, Article ID 9060852, 5 pages, 2017. doi:10.1155/2017/9060852
9. Cotterill, S, Rowland, AG, Kelly, J, Lees, H and Kamara, M 2016, 'Diagnostic accuracy of PAT-POPS and ManChEWS for admissions of children from the emergency department', Emergency Medicine Journal, 33 (11)
10. Challen K and Roland D. Early Warning Scores: a health warning EMJ 2016, 33(11):812-817
11. Riaz, S, Rowland, AG, Woby, SR, Long, T, Livesley, J, Cotterill, S, Heal, C and Roland, D 2018, 'Refining and testing the diagnostic accuracy of an assessment tool (PAT-POPS) to predict admission and discharge of children and young people who attend an emergency department : protocol for an observational study' , BMC Pediatrics, 18 (303)
12. StataCorp. 2015. Stata Statistical Software: Release 14. College Station, TX: StataCorp LP.
13. Moons KG, Altman DG, Reitsma JB, et al. Transparent Reporting of a multivariable prediction model for Individual Prognosis Or Diagnosis (TRIPOD): Explanation and Elaboration. Ann Intern Med. 2015;162:W1–W73. doi: https://doi.org/10.7326/M14-0698
14. Cohen JF, Korevaar DA, Altman DG, et al STARD 2015 guidelines for reporting diagnostic accuracy studies: explanation and elaboration BMJ Open 2016;6:e012799. doi: 10.1136/bmjopen-2016-012799
15. Kalton, G., & Kish, L. (1981). Two efficient random imputation procedures. In Proceedings of the survey research methods section (pp. 146-151). American Statistical Association
16. Royston P, Sauerbrei W. Multivariable model-building: a pragmatic approach to regression anaylsis based on fractional polynomials for modelling continuous variables. John Wiley & Sons; 2008 Sep 15
17. Wolff RF, Moons KG, Riley RD, et al, for the PROBAST Group†. PROBAST: A Tool to Assess the Risk of Bias and Applicability of Prediction Model Studies. Ann Intern Med. 2019;170:51–58. doi: https://doi.org/10.7326/M18-1376
18. Riley, R. D., Ensor, J., Snell, K. I., Debray, T. P., Altman, D. G., Moons, K. G., & Collins, G. S. (2016). External validation of clinical prediction models using big datasets from e-health records or IPD meta-analysis: opportunities and challenges. bmj, 353, i3140
19. Sullivan, L.M., Massaro, J.M. and D'Agostino Sr, R.B., 2004. Presentation of multivariate data for clinical use: The Framingham Study risk score functions. Statistics in medicine, 23(10), pp.1631-1660.

For peer review only - http://bmjopen.bmj.com/site/about/guidelines.xhtml

20. This data was obtained via a Freedom of Information request to NHS Digital but did not provide confidence intervals or a range
21. Seiger N, Maconochie I, Oostenbrink R, Moll HA. Validity of different pediatric early warning scores in the emergency department. Pediatrics. 2013;132(4):e841–e850. doi:10.1542/peds.2012-3594

For peer review only

For peer review only - http://bmjopen.bmj.com/site/about/guidelines.xhtml

Table 1: Characteristics of the sample population at each site (who met the inclusion criteria)

	Site 1 (Emergency Department) (development dataset) Visits = 21480 Participants = 15484			Site 2 (Emergency Department) Visits = 9972 Participants = 7817			Site 3 (Urgent Care Centre) Visits = 13429 Participants = 9971		
Variable	Summary	Min/Max	Missing (%)	Summary	Min/Max	Missing (%)	Summary	Min/Max	Missing (%)
**Age** in years (median, IQR)	3 (1 to 8)	0/15	0	7 (3 to 12)	0/15	0	6 (2 to 10)	0/15	0
**Gender**						0			0
Female	9384 (44%)		0	4486 (45%)			6131 (54%)		
**Ethnicity**			0			0			0
White British	11162 (52%)			7081 (71%)			7046 (52%)		
Irish + Gypsy or Irish Traveller	34 (0%)			26 (0%)			61 (0%)		
Other White	569 (3%)			298 (3%)			369 (3%)		
White and Black Caribbean	158 (1%)			89 (1%)			51 (0%)		
White and Black African	121 (1%)			53 (1%)			80 (1%)		
White and Asian	255 (1%)			118 (1%)			217 (2%)		
Other Mixed	294 (1%)			107 (1%)			244 (2%)		
Indian	75 (0%)			45 (0%)			51 (0%)		
Pakistani	4245 (20%)			1165 (12%)			61 (0%)		
Bangladeshi	2060 (10%)			36 (0%			524 (4%)		
Chinese	46 (0%)			42 (0%)			29 (0%)		
Other Asian + Asian/Asian British	302 (1%)			202 (2%)			213 (2%)		
African									
Caribbean	374 (2%)			114 (1%)			242 (2%)		
Other Black +	24 (0%)			8 (0%)			21 (0%)		
Black/African/Caribbean/Black	149 (1%)			54 (1%)			168 (1%)		
British									
Unknown / not stated	1010 (5%)			270 (3%)			169 (1%)		
**Heart rate** (mean /sd)	121.6 (30.5)		11	104.5 (26.1)		4	109.7 (27.0)		2
**Temperature** (median /iqr)	36.9 (36.5 to 37.4)	32.6/41.7	10	36.7 (36.4 to 37.1)	34.4/41.1	3	36.7 (36.4 to 37.1)	35.0/41.2	1

For peer review only

For peer review only - http://bmjopen.bmj.com/site/about/guidelines.xhtml

	Site 1 (Emergency Department) (development dataset) Visits = 21480 Participants = 15484			Site 2 (Emergency Department) Visits = 9972 Participants = 7817			Site 3 (Urgent Care Centre) Visits = 13429 Participants = 9971		
**Respiratory rate**	26 (22 to 32)	10/90**	10	22 (20 to 26)	12/88	3	24 (20 to 26)	12/80	2
**Oxygen saturation**	99 (98 to 100)	63/100	10	99 (98 to 100)	70/100	4	99 (99 to 100)	67/100	2
**Requires supplement oxygen**	386 (2%)		0	31 (0%)		0	99 (1%)		0
**Breathing**			2			0			0
None of the above	19678 (93%)			9713 (98%)			13038 (98%)		
Audible grunt	52 (0%)			9 (0%)			25 (0%)		
Wheeze	709 (3%)			135 (1%)			190 (1%)		
Stridor	52 (0%)			6 (0%)			9 (0%)		
Tracheal tug	624 (3%)			68 (1%)			102 (1%)		
**Recession**			0			0			0
No recession	18757 (87%)			9748 (98%)			12900 (96%)		
Mild recession	1764 (8%)			153 (2%)			338 (3%)		
Moderate	743 (3%)			43 (0%)			114 (1%)		
Severe	213 (1%)			27 (0%)			75 (1%)		
**Responsiveness**			0			0			0
Alert	21044 (98%)			9775 (98%)			13252 (99%)		
Responds to pain	53 (0%)			10 (0%)			5 (0%)		
Responds to voice	126 (1%)			15 (0%)			19 (0%)		
Unresponsive	253 (1%)			171 (2%)			151 (1%)		
**Nurse Judgement**			0			0			0
No concern	6476 (30%)			6894 (69%)			9888 (74%)		
Low level concern	11645 (54%)			2756 (28%)			3268 (24%)		
Child looks unwell/high concern	3355 (16%)			321 (3%)			271 (2%)		
**Behaviour**			0			0			0
Normal for age	20349 (95%)			9720 (97%)			13105 (98%)		
Agitated	356 (2%)			105 (1%)			121 (1%)		
Floppy	138 (1%)			22 (0%)			23 (0%)		
Inappropriate	54 (0%)			9 (0%)			8 (0%)		
Listless	579 (3%)			115 (1%)			170 (1%)		

For peer review only - http://bmjopen.bmj.com/site/about/guidelines.xhtml

	**Site 1 (Emergency Department) (development dataset) Visits = 21480 Participants = 15484**			**Site 2 (Emergency Department) Visits = 9972 Participants = 7817**			**Site 3 (Urgent Care Centre) Visits = 13429 Participants = 9971**		
Multi-morbidity	749 (3%)		0	148 (1%)		0	551 (4%)		0
**Arrived by ambulance**	4539 (21%)		0	20 (0%)		0	50 (0%)		0
**Admitted now or next 7 days**	6810 (32%)		0	600 (6%)		0	1009 (8%)		0

For peer review only

For peer review only - http://bmjopen.bmj.com/site/about/guidelines.xhtml

Table 2: Final regression model

Variable	OR	95% CI low	95% CI high	P
Age				<0.001
0-1 months	1.880	1.876	1.885	
2-5 months	1.279	1.276	1.282	
6-11 months	1.125	1.122	1.282	
1-3 years	1.031	1.029	1.034	
4-16 years	Reference			
Heart rate				
<75	.959	.807	1.141	0.638
75-125	Reference			
>125	1.422	1.303	1.552	<0.001
Temperature (degrees Celsius)				<0.001
<38	Reference			
38-39	1.365	1.307	1.424	
>39	1.763	1.689	1.840	
Respiratory rate	1.029	1.023	1.035	<0.001
Oxygen saturation				<0.001
95-100	Reference			
90-94	3.381	2.654	4.305	
<90	5.477	2.953	10.157	
Requires supplementary oxygen	1.872	1.437	2.440	<0.001
Breathing				
Normal	Reference			
Abnormal	1.681	1.423	1.985	<0.001
Recession				
None	Reference			
Mild	1.281	1.117	1.469	<0.001
Moderate/Severe	1.484	1.183	1.863	0.001
Behaviour category				
Normal	Reference			
Agitated	1.353	1.043	1.754	0.023
Floppy	2.046	1.371	3.054	<0.001
Listless	1.498	1.217	1.843	<0.001
Nurse judgement				
No concern	Reference			
Low level concern	1.361	1.258	1.473	<0.001
High concern	2.677	2.388	3.002	<0.001
Multimorbidity	1.937	1.599	2.346	<0.001
Arrived by ambulance	2.416	2.223	2.625	<0.001
Advised by medical professional	2.159	1.975	2.361	<0.001
Constant	.144	0.133	0.156	<0.001

For peer review only

For peer review only - http://bmjopen.bmj.com/site/about/guidelines.xhtml

Table 3: external validation results

Site	Briers score	Calibration slope	CITL	E/O	C-index
2	0.065	0.986<br>(0.897 to 1.076)	-1.219<br>(-1.306 to -1.132)	2.673	0.763<br>(0.742 to 0.783)
3	0.073	1.075<br>(1.002 to 1.149)	-1.055<br>(-1.123 to -.987)	2.275	0.753<br>(0.737 to 0.770)

For peer review only

For peer review only - http://bmjopen.bmj.com/site/about/guidelines.xhtml

Table 4: PAGE point scoring tool

Variable	Category	Point(s)
Age (months)	0-1	2
	2-5	1
	≥ 6	0
Heart rate (BPM)	≤ 125 (ie 0-125)	0
	>125 (ie 126 and above)	1
Respiratory rate	0-25	0
	26-60	2
	>60	3
Temperature (degrees Celsius)	<38	0
	38-39	1
	>39	2
Oxygen saturation (%)	>94	0
	90-94	4
	<90	5
Requires supplementary oxygen	No	0
	Yes	2
Breathing	Normal	0
	Abnormal	2
Recession	No recession	0
	Any recession	1
Behaviour	Normal	0
	Agitated or Listless	1
	Floppy	2
Nurse Judgement	No concern	0
	Low level concern	1
	High concern	3
Multi-morbidity	No	0
	Yes	2
Arrived by ambulance	No	0
	Yes	3
Advised by medical professional to attend	No	0
	Yes	2

For peer review only

For peer review only - http://bmjopen.bmj.com/site/about/guidelines.xhtml

Table 5: Sensitivity and specificity for each cut-point of PAGE

Cut-point for admission decision	Sensitivity (95% CI)	Specificity (95% CI)	Likelihood ratio +	Likelihood ratio -	Positive predictive value	Negative predictive value
≥0	100%	0%	1.0000	-	-	-
≥1	96.83 (96.38 to 97.23)	15.80 (15.22 to 16.41)	1.1501	0.2010	34.8 (34.2 to 35.4)	91.5 (91.1 to 91.9)
≥2	91.13 (90.42 to 91.79)	36.34 (35.56 to 37.13)	1.4315	0.2441	39.9 (39.3 to 40.6)	89.8 (89.4 to 90.2)
≥3	87.70 (86.89 to 88.46)	45.84 (45.03 to 46.65)	1.6192	0.2684	42.9 (42.3 to 43.6)	88.9 (88.5 to 89.3)
≥4	78.87 (77.90 to 79.81)	61.03 (62.23 to 63.82)	2.1330	0.3353	48.5 (47.8 to 49.1)	85.7 (85.2 to 86.2)
≥5	68.61 (67.49 to 69.70)	73.69 (72.97 to 74.40)	2.6074	0.4261	54.8 (54.1 to 55.4)	83.5 (83.0 to 84.0)
≥6	59.47 (58.29 to 60.64)	80.86 (80.21 to 81.49)	3.1070	0.5012	59.1 (58.4 to 59.7)	81.1 (80.6 to 81.7)
≥7	48.56 (47.37 to 49.76)	87.89 (87.03 to 88.11)	3.9099	0.5873	64.5 (63.8 to 65.1)	78.6 (78.0 to 79.1)
≥8	38.88 (37.73 to 40.06)	92.20 (91.75 to 92.62)	4.9819	0.6629	69.8 (69.2 to 70.4)	76.5 (75.9 to 77.0)
≥9	29.84 (28.76 to 30.94)	95.34 (94.99 to 95.68)	6.4089	0.7359	74.8 (74.3 to 75.4)	74.5 (74.0 to 75.1)
≥10	22.11 (21.14 to 23.12)	97.43 (97.16 to 97.68)	8.6053	0.7994	80.0 (79.4 to 80.5)	72.9 (72.3 to 73.5)
≥11	16.36 (15.49 to 17.26)	98.56 (98.35 to 98.75)	11.3733	0.8486	84.1 (83.6 to 84.6)	71.7 (71.1 to 72.3)
≥12	12.03 (11.27 to 12.83)	99.28 (99.12 to 99.41)	16.6441	0.8861	88.5 (88.1 to 89.0)	70.9 (70.3 to 71.5)
≥13	8.33 (7.69 to 9.01)	99.60 (99.48 to 99.69)	20.7021	0.9204	90.6 (90.2 to 91.0)	70.1 (69.5 to 70.7)
≥14	5.49 (4.97 to 6.07)	99.79 (99.70 to 99.85)	25.9892	0.9471	92.4 (92.0 to 92.7)	69.5 (68.9 to 70.1)
≥15	3.25 (2.84 to 3.70)	99.85 (99.77 to 99.90)	21.6398	0.9690	91.0 (90.6 to 91.3)	69.0 (68.4 to 69.6)
≥16	2.14 (1.82 to 2.52)	99.90 (99.83 to 99.94)	20.9674	0.9796	90.7 (90.3 to 91.1)	68.7 (68.1 to 69.4)
≥17	1.12 (0.89 to 1.40)	99.97 (99.92 to 99.99)	32.7436	0.9867	93.8 (93.5 to 94.2)	68.5 (67.9 to 69.2)
≥18	0.65 (0.48 to 0.88)	100 (99.97 to 100)	-	0.9935	100.0 (100.0 to 100.0)	68.4 (67.8 to 69.1)
≥19	0.34 (0.22 to 0.52)	100 (99.97 to 100)	-	0.9966	100.0 (100.0 to 100.0)	68.4 (67.8 to 69.0)
≥20	0.12 (0.06 to 0.24)	100 (99.97 to 100)	-	0.9988	100.0 (100.0 to 100.0)	68.3 (67.7 to 68.9)
≥21	0.04 (0.01 to 0.14)	100 (99.97 to 100)	-	0.9996	100.0 (100.0 to 100.0)	68.3 (67.7 to 68.9)
≥22	0.02 (0.00 to 0.10)	100 (99.97 to 100)	-	0.9996	100.0 (100.0 to 100.0)	68.3 (67.7 to 68.9)
≥23	0	100	-	1.0000	100.0 (100.0 to 100.0)	68.3 (67.7 to 68.9)

For peer review only - http://bmjopen.bmj.com/site/about/guidelines.xhtml

For peer review only

For peer review only - http://bmjopen.bmj.com/site/about/guidelines.xhtml

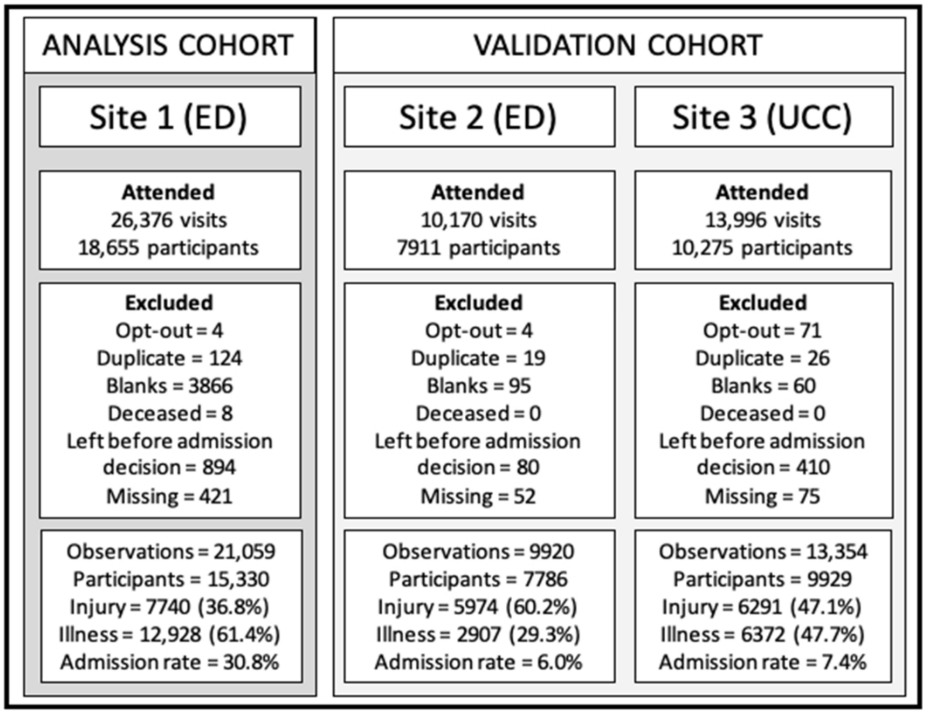

Figure 1 - Development of the dataset

254x190mm (300 x 300 DPI)

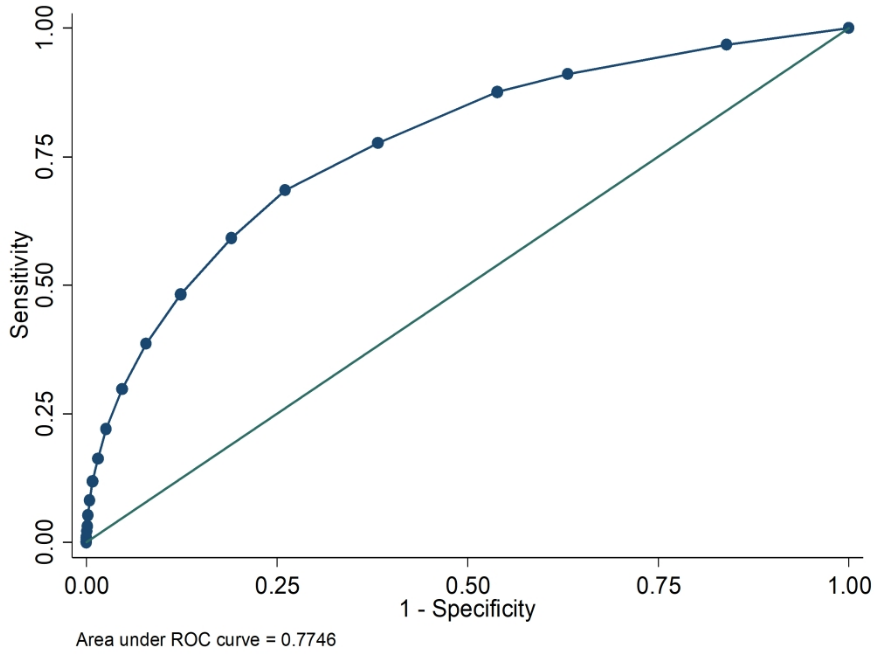

Figure 2 Area under the ROC curve of the PAGE scoring tool

254x190mm (300 x 300 DPI)

Statistical Appendix

This appendix contains only details not included in the main paper. It is intended that this Appendix will be made available online-only as a supplementary file.

Use of variables in the model

All of the variables were used in the model with the form of the individual variables and their suitability for inclusion in the model considered and adjusted as necessary.

Categorical variables with small categories

The category of behaviour 'inappropriate' was recorded for 54 children (<1%). This was too small a category to include in the modelling, and it was not clear what other category to amalgamate it with, as both 'floppy' and 'listless' could be considered 'inappropriate'. These were marked as missing, removing those children from the analysis. The four categories of abnormal breathing (audible grunt, wheeze, stridor and tracheal tug) were very small (0 to 3%) and so breathing was re-categorised as 'normal' and 'abnormal'. The category 'severe recession' was recorded for only 311 children over the three sites (between 0 and 1% per site), so 'severe' and 'moderate' recession were combined.

Continuous variables

During the regression modelling the fractional polynomial command made suggestions for the transformation of continuous variables. Age was included as reciprocal fractional polynomial of the original variable. Temperature and respiratory

For peer review only - http://bmjopen.bmj.com/site/about/guidelines.xhtml

rate had a linear relationship with admittance and were included in the model as continuous variables.

For heart rate and oxygen saturation the plots of the fractional polynomials laid over the data were examined to visually assess how the models fit. These plots and the findings from the exploratory analysis jointly led decisions on the ultimate form of each of the variables.

- **Heart rate:** The fractional polynomial suggested a linear association with admittance. Examining the plot revealed that although a linear relationship is broadly appropriate it does a poor job of capturing the two large dips in probability of admittance. A more complex plot would have captured better the association between heart rate and admittance. With the large sample size such a curve could have been fitted without being overly concerned about overfitting. However, this would have defeated the overall aim of an easily used paper-based point scoring tool by requiring multiple coefficients in the final model. Heart rate was categorised as under 75, 75 to 125 and over 125.
- **Oxygen saturation:** The multivariable fractional polynomial modelling suggested that the raw oxygen saturation variable taken to the power of -2 was the best fitting form. On examination of the fractional polynomial plot it was clear that the plot fitted the data very well for oxygen saturations up to 90%, but above 90%, where 99% of the data lay, the polynomial failed to capture the more erratic association between saturation and admittance. Accordingly, a decision was taken to categorise the oxygen saturation variable based on the frequencies of the responses at each saturation

For peer review only - http://bmjopen.bmj.com/site/about/guidelines.xhtml

percentage, its observed relationship with admittance from the graph and on the previous PAT-POPS model (which aligns with clinical opinion of oxygen saturation cut-offs): 95%-100%; 90%-94%; <90%.

Being aware of the statistical implications of categorising continuous variables these decisions were not made lightly, however it was clear that no fractional polynomial would be able to capture the nature of the relationship better than categorisation.

Internal validation

Figure A shows the internal validation calibration plot. The blue ‘lowess’ line fitting the reference line so well is a demonstration of how well the model is fitting the data. Given the large sample size however this is to be expected. **Table A** shows the various internal validation results. The ‘apparent’ results are the average measures of validation for the 500 models created from the 500 bootstraps of the data. The ‘test’ results show the average performance of the models developed for each of those 500 bootstraps when applied to the original dataset. The ‘optimism’ results are the ‘apparent’ results subtracted from the ‘test’ results. ‘test’ results similar to ‘apparent’ results (and subsequently small ‘optimism’ results) suggest strong internal validity.

For peer review only - http://bmjopen.bmj.com/site/about/guidelines.xhtml

Figure A. Calibration plot for internal validation.

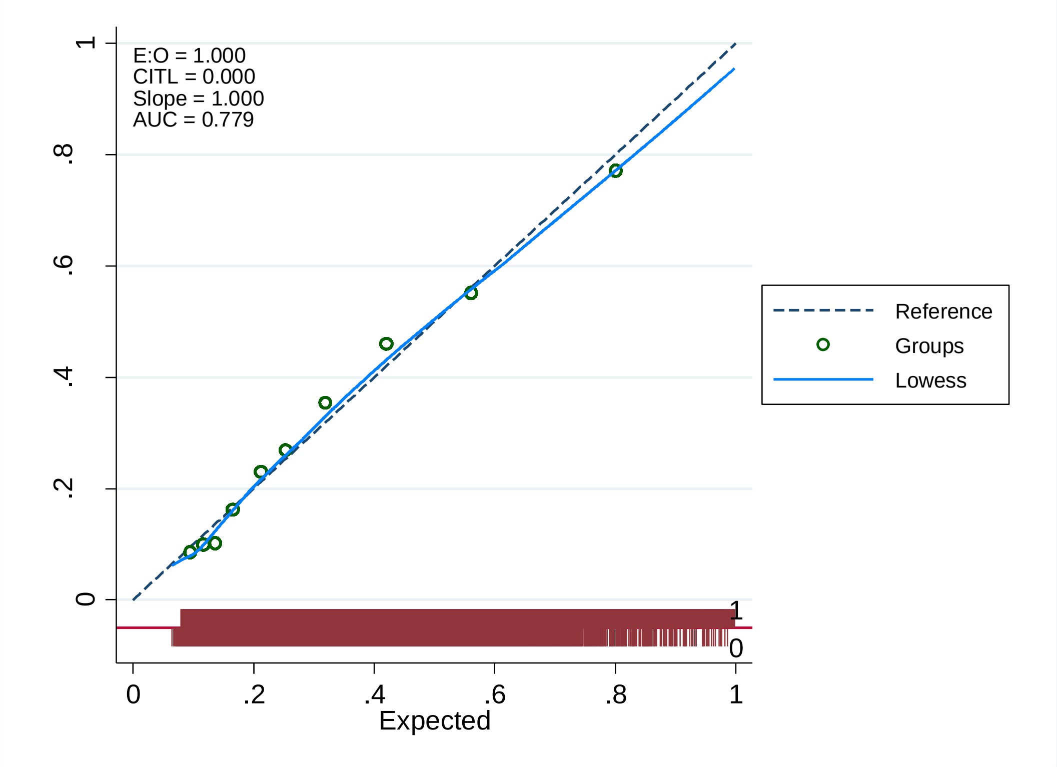

Table A. Results of internal validation.

Measure	Bootstraps	Mean	Standard deviation	Minimum	Maximum
**Apparent C-index**	500	.7790287	.0035788	.7685792	.7889659
**Apparent CITL**	500	0.00000000393	0.0000000181	-0.000000103	0.000000190
**Apparent slope**	500	1	0.0000000549	1	1.000001
**Test C-index**	500	.7780273	.0005261	.7751072	.7791697
**Test CITL**	500	.0000289	.0158041	-.0458299	.0523462
**Test slope**	500	.9934335	.0175813	.9502897	1.043822
**Optimism C-index**	500	.0010013	.0035684	-.0097837	.0107504
**Optimism CITL**	500	-.0000289	.0158041	-.0523462	.0458299
**Optimism slope**	500	.0065665	.0175813	-.0438216	.0497103

For peer review only - http://bmjopen.bmj.com/site/about/guidelines.xhtml

External validation: Site 2

Model reproducibility or transportability

The C-index for this membership model was 0.8078. The closer this number is to 1 the more likely it is that this represents model transportability rather than model reproducibility. So, in carrying out external validation using the Site 2 dataset an assessment is being made of whether the model works well in a different but related population. This was as expected given that from the exploratory analysis it could be seen that Site 2 represent a different mix of ethnicities as well as generally older and healthier patients.

Calibration and discrimination

The Brier score was lower in the Site 2 data (0.0653) than in the model development dataset, suggesting very good prediction accuracy in this external dataset. At 0.9864908 (0.896986 to 1.075996) the calibration slope was also close to the ideal of 1. Calibration-in-the-large (CITL) was less impressive at -1.218977 (-1.305886 to -1.132068). This suggests that the predicted probabilities were higher than the observed proportions. So, children in Site 2 were less likely to be admitted than the model predicted; a child with equivalent characteristics presenting at Site 1, for example, would be more likely to be admitted. This can be seen in the calibration plot **(Figure B)** where the observed probabilities are consistently lower than the expected probabilities, especially as the expected probabilities near 1. This was also evident in the E/O ratio of 2.7. This is the expected/observed ratio, how many were expected to be admitted based on the model versus how many actually were

For peer review only - http://bmjopen.bmj.com/site/about/guidelines.xhtml

admitted, so ideally this would be 1. That it is higher here suggests less people were admitted than would have been expected from the model. In terms of discrimination, the C-index was 0.7626 (0.74244 to 0.78279) **(Figure B)**.

Figure B. Calibration plot for Site 2 external validation.

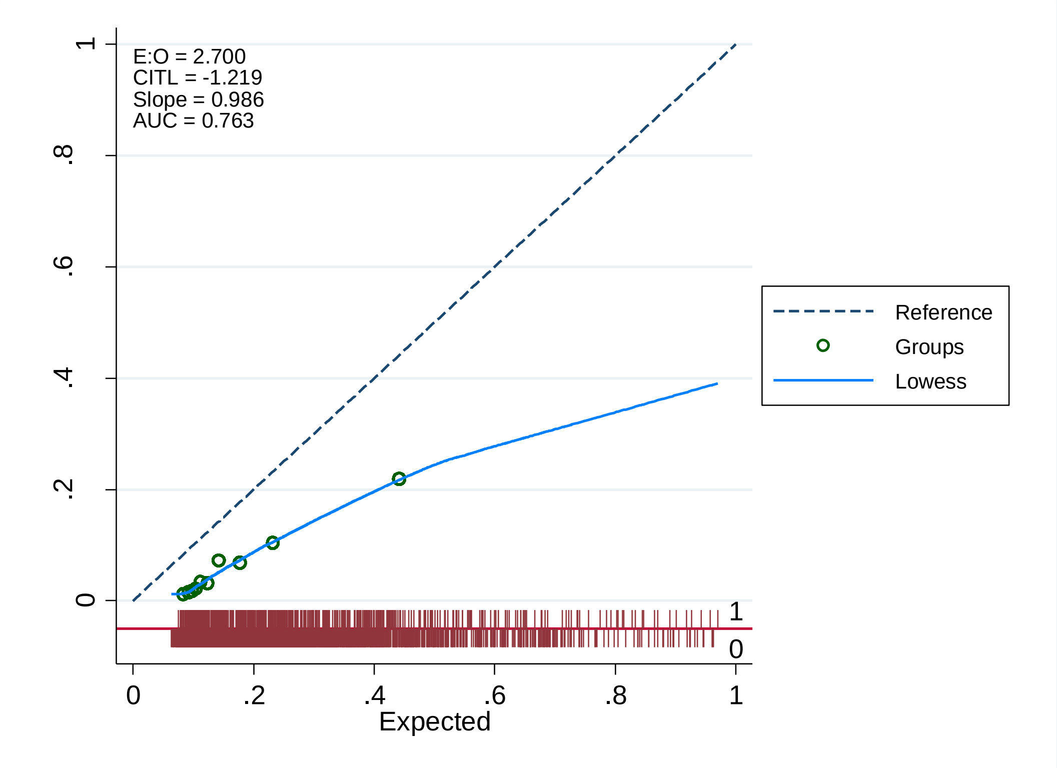

External validation: Site 3

Model reproducibility of transportability

The C-index for this membership model was 0.8154. This suggests that Site 1 and Site 3 are also quite different from each other in terms of their populations and therefore model transportability was assessed with the following results. That these two sites differ is expected; aside from the difference in ethnic make-up of the populations, Site 1 is an ED whereas Site 3 is an Urgent care centre and therefore sees healthier children.

For peer review only - http://bmjopen.bmj.com/site/about/guidelines.xhtml

Calibration and discrimination

As with Site 2, when the model was applied to Site 3 the Brier score was a very low 0.0732. The calibration slope was over 1 at 1.075, this time suggesting overfitting, but as with Site 2 the CI also crossed 1 (0.976 to 1.123). The CITL was -1.055 (-1.123 to -0.987) suggesting predicted probabilities were higher than the observed proportions, again evident in **Figure C**. The E/O ratio was 2.3. The C-index was 0.7533 (0.734 to 0.770).

Figure C. Calibration plot for Site 3 external validation.

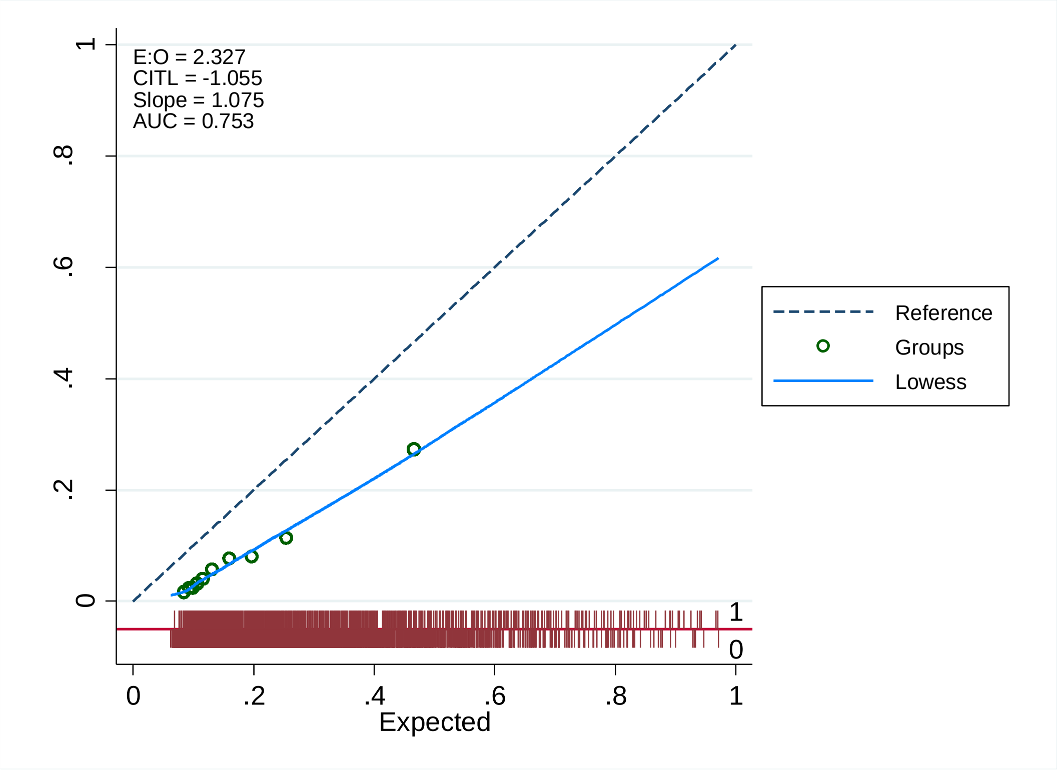

Overall

From these results it can be determined that the model transportability is good overall. With only slight over/under-fitting present at each of the external validation sites and C-indexes only modestly smaller than was found in the original model. It is

For peer review only - http://bmjopen.bmj.com/site/about/guidelines.xhtml

clear however that the model may over-estimate the probability of admission in external populations, probably owing to the admission rate at the development site (Site 1).

For peer review only

For peer review only - http://bmjopen.bmj.com/site/about/guidelines.xhtml

TRIPOD Checklist: Prediction Model Development

Section/Topic	Item	Checklist Item	Page
**Title and abstract**			
Title	1	Identify the study as developing and/or validating a multivariable prediction model, the target population, and the outcome to be predicted.	1
Abstract	2	Provide a summary of objectives, study design, setting, participants, sample size, predictors, outcome, statistical analysis, results, and conclusions.	3-4
**Introduction**			
Background and objectives	3a	Explain the medical context (including whether diagnostic or prognostic) and rationale for developing or validating the multivariable prediction model, including references to existing models.	5-6
	3b	Specify the objectives, including whether the study describes the development or validation of the model or both.	6
**Methods**			
Source of data	4a	Describe the study design or source of data (e.g., randomized trial, cohort, or registry data), separately for the development and validation data sets, if applicable.	6-7
	4b	Specify the key study dates, including start of accrual; end of accrual; and, if applicable, end of follow-up.	6
Participants	5a	Specify key elements of the study setting (e.g., primary care, secondary care, general population) including number and location of centres.	6-7
	5b	Describe eligibility criteria for participants.	6-7
	5c	Give details of treatments received, if relevant.	–
Outcome	6a	Clearly define the outcome that is predicted by the prediction model, including how and when assessed.	7
	6b	Report any actions to blind assessment of the outcome to be predicted.	–
Predictors	7a	Clearly define all predictors used in developing or validating the multivariable prediction model, including how and when they were measured.	7
	7b	Report any actions to blind assessment of predictors for the outcome and other predictors.	–
Sample size	8	Explain how the study size was arrived at.	8
Missing data	9	Describe how missing data were handled (e.g., complete-case analysis, single imputation, multiple imputation) with details of any imputation method.	13-14
Statistical analysis methods	10a	Describe how predictors were handled in the analyses.	9
	10b	Specify type of model, all model-building procedures (including any predictor selection), and method for internal validation.	9-10
	10d	Specify all measures used to assess model performance and, if relevant, to compare multiple models.	10-11
Risk groups	11	Provide details on how risk groups were created, if done.	–
**Results**			
Participants	13a	Describe the flow of participants through the study, including the number of participants with and without the outcome and, if applicable, a summary of the follow-up time. A diagram may be helpful.	Fig 1
	13b	Describe the characteristics of the participants (basic demographics, clinical features, available predictors), including the number of participants with missing data for predictors and outcome.	Table 1
Model development	14a	Specify the number of participants and outcome events in each analysis.	Fig 1
	14b	If done, report the unadjusted association between each candidate predictor and outcome.	–
Model specification	15a	Present the full prediction model to allow predictions for individuals (i.e., all regression coefficients, and model intercept or baseline survival at a given time point).	Table 2
	15b	Explain how to the use the prediction model.	17
Model performance	16	Report performance measures (with CIs) for the prediction model.	15-16
**Discussion**			
Limitations	18	Discuss any limitations of the study (such as nonrepresentative sample, few events per predictor, missing data).	19
Interpretation	19b	Give an overall interpretation of the results, considering objectives, limitations, and results from similar studies, and other relevant evidence.	17-19
Implications	20	Discuss the potential clinical use of the model and implications for future research.	20
**Other information**			
Supplementary information	21	Provide information about the availability of supplementary resources, such as study protocol, Web calculator, and data sets.	4, 15
Funding	22	Give the source of funding and the role of the funders for the present study.	~~1~~ 2

We recommend using the TRIPOD Checklist in conjunction with the TRIPOD Explanation and Elaboration document.
